# Insights on Dietary Polyphenols as Agents against Metabolic Disorders: Obesity as a Target Disease

**DOI:** 10.3390/antiox12020416

**Published:** 2023-02-08

**Authors:** Simon-Okomo Aloo, Fred Kwame Ofosu, Nam-Hyeon Kim, Sheila M. Kilonzi, Deog-Hwan Oh

**Affiliations:** 1 Department of Food Science and Biotechnology, College of Agriculture and Life Sciences, Kangwon National University, Chuncheon 24341, Republic of Korea; 2 Department of Food Science and Nutrition, School of Agriculture and Biotechnology, Karatina University, Karatina 10101, Kenya

**Keywords:** anti-obesity, bioavailability, functional ingredient, gut microbiota, safety

## Abstract

Obesity is a condition that leads to increased health problems associated with metabolic disorders. Synthetic drugs are available for obesity treatment, but some of these compounds have demonstrated considerable side effects that limit their use. Polyphenols are vital phytonutrients of plant origin that can be incorporated as functional food ingredients. This review presents recent developments in dietary polyphenols as anti-obesity agents. Evidence supporting the potential application of food-derived polyphenols as agents against obesity has been summarized. Literature evidence supports the effectiveness of plant polyphenols against obesity. The anti-obesity mechanisms of polyphenols have been explained by their potential to inhibit obesity-related digestive enzymes, modulate neurohormones/peptides involved in food intake, and their ability to improve the growth of beneficial gut microbes while inhibiting the proliferation of pathogenic ones. Metabolism of polyphenols by gut microbes produces different metabolites with enhanced biological properties. Thus, research demonstrates that dietary polyphenols can offer a novel path to developing functional foods for treating obesity. Upcoming investigations need to explore novel techniques, such as nanocarriers, to improve the content of polyphenols in foods and their delivery and bioavailability at the target sites in the body.

## 1. Introduction

**Figure 1:**
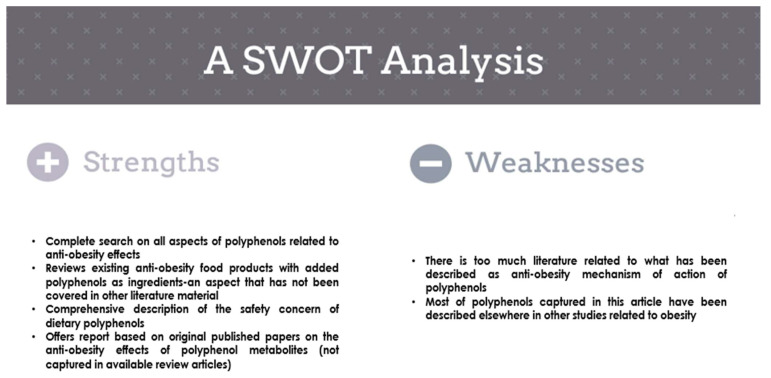
Strengths and weaknesses of the current article.

Obesity results from the excessive accumulation of body fat due to an imbalance between energy intake and expenditure. Obesity is a risk factor for several other chronic disorders, including type 2 diabetes mellitus, cardiovascular diseases, stroke, dementia, osteoarthritis, and breast, colon, and prostate cancers [1,2]. It can contribute to a decline in life quality and expectancy and may also lead to many other psychological problems. Modifying lifestyle is considered the most appropriate approach to reducing obesity [1]. In particular, adjusting an individual’s diet to include healthier foods is vital in weight management. Globally, obesity is constantly growing in prevalence and significance, posing a threat to health. Once just a problem of wealthy nations, obesity now impacts more countries at all economic levels, bringing with it a wave of ill health and loss of productivity among the population. The worldwide prevalence of the disease has doubled since the beginning of 1980 [3]. Today, obesity is considered one of the most serious public health concerns, with the latest figures from the World Health Organization’s database showing that about one in ten young people, ages 5–17, are overweight or obese, while the condition is even severe among middle-aged people [3]. Bariatric surgery and synthetic drugs (such as orlistat and naltrexone/bupropion, glucagon-like peptide-1 receptor agonists) have been accepted as therapeutic strategies for obesity. However, these treatment methods have been associated with unpleasant side effects that limit their use [4]. As a result of these concerns, other alternative approaches are needed to help fill this gap and reduce the impact of obesity among the population. This challenge has led to more research on obesity, including assessing the effect of polyphenols on disease. Polyphenols are vital phytonutrients of plant origin that can be incorporated as functional ingredients in foods to offer an effective natural therapy against obesity. There have been efforts to discover alternative obesity treatments from dietary polyphenols [1,2]. Although several other papers have been on this subject, the current article reviewed the most current knowledge on the relationship between plant-derived polyphenols and obesity. The cellular, animal, and human data supporting the anti-obesity potential of phenolic compounds have been summarized. The information presented in the review is unique in revealing the potential role of polyphenols in weight management and their safety-related issues. The article offers the most comprehensive evidence, combining the incorporation of polyphenols into foods, safety aspects, bioavailability, and related research limitations in preclinical and clinical studies in an attempt to provide a lead for more studies regarding foods targeting obesity. However, this article has some weaknesses as described in Figure 1.

## 2. Existing Treatment for Obesity and Their Safety Limitations

The existing methods of obesity management include surgical procedures, administration of synthetic drugs, and diet and exercise. The combination of diet and exercise is considered the safest and most appropriate method for weight management. Bariatric surgery refers to various surgical procedures that alter the anatomy of the gastrointestinal tract [4]. Even though it is an effective method of obesity treatment, the cost of bariatric surgery is high, and its post-surgery care is a burden to most patients [4]. In addition, the procedure is associated with various risks of developing acute kidney injury, marginal ulcers, and gallstones [4].

On the other hand, drug therapy is an adjunct to diet and exercise in people who require more advanced intervention. The current anti-obesity drugs aim to reduce food intake by either curbing appetite or suppressing the craving for food. Conventional anti-obesity drugs exert anorectic action due to their effects on the levels of monoamines in various brain nuclei [5]. However, in addition to their anorexic effects, these drugs have been linked to altered brain functions associated with sexual behavior, hormonal secretion, mood, and sleep, among other neuro-related effects [5]. In addition to associated neuro influences, some of these compounds, such as orlistat, have been linked with gastrointestinal and cardiovascular side effects such as steatorrhoea, fecal incontinence, flatulence, and malabsorption of fat-soluble vitamins, making their use limited [5]. To overcome the challenges arising from the use of orlistat, a new pancreatic lipase inhibitor, cetilistat, was developed [6]. Cetilistat has been found to show considerable potential in the treatment of obesity; nonetheless, evidence from in vivo studies indicates that the gastrointestinal effects can persist even with the use of cetilistat [6]. 

There is also ongoing research on developing anti-obesity treatments based on the two incretin hormones: glucose-dependent insulinotropic polypeptide (GIP) and glucagon-like peptide-1 (GLP-1). Treating diseases by administering agonists of GIP and GLP-1 exploits their physiological effects on the body [7]. These compounds may demonstrate anti-obesity effects by engaging endogenous hormones involved in energy balance and metabolism [7], although they, too, have several limitations (Table 1). Consequently, there is a substantial unmet need to find effective and safer anti-obesity management methods; research is still needed on natural plant metabolites such as polyphenols to help understand their anti-obesity efficacy

## 3. Diversity of Polyphenols in Diet and Their Biological Significance

**Figure 2:**
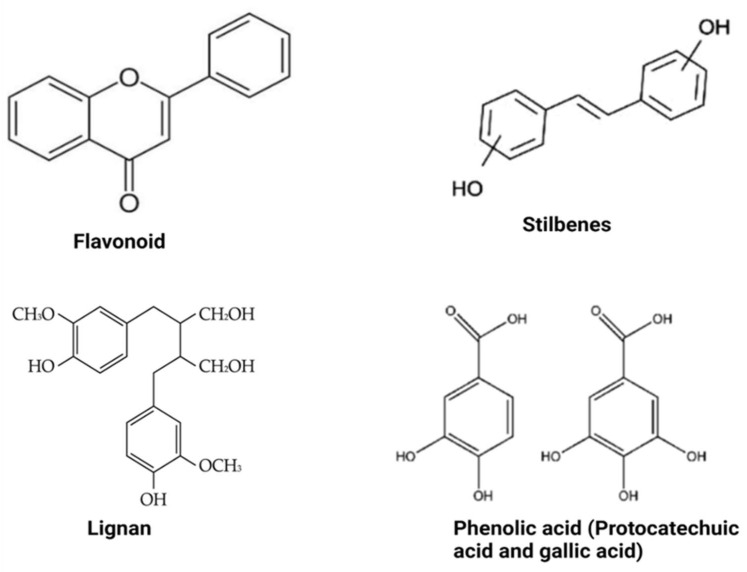
Structures of flavonoids and Non-flavonoids polyphenols.

Polyphenols are compounds with several hydroxyl groups on an aromatic ring. Most of these compounds can be found in several plant foods, including tea leaves, cereals, legumes, and fruits, while some are mainly associated with specific foods (e.g., isoflavones in soya, phloridzin in apples, flavanones in citrus, etc.). They can be grouped into two broad categories: flavonoid-type and non-flavonoid-types with distinct structures (Figure 2). These groups are discussed below.

### 3.1. Flavonoid-Type Phenolic Compounds

Flavonoid-type phenolic compounds are further grouped into subclasses of flavanols, flavonols, isoflavones, flavanones, anthocyanins, and proanthocyanidins. **Flavonols** are the groups of polyphenols containing a 3-hydroxyflavone base (3-hydroxy-2-phenylchromen-4-one) on their structure. They are distinguished from other polyphenols by hydroxy modification at distinct positions of the phenol residue [22]. Foods with specified amounts of these compounds but lacking other active ingredients have demonstrated strong anti-obesity effects [23]. Green tea rich in flavonols may reduce obesity through their thermogenic effect and increased fat oxidation [23]; they can also inhibit enzymes involved in fat synthesis [23]. **Flavones** have a basic structure consisting of a 2-phenyl-benzo-γ-pyrone skeleton formed by two phenyl rings (A and B) linked with a heterocyclic pyrone ring [22]. They are said to have relatively higher bioavailability in the body compared to flavonols, probably because of degradation by gut microbiota and greater accessibility for absorption in the intestine [22]. Flavones such as ellagitannins and granatin B present in the edible flowers of *Punica granatum* L. were reported to exert a potent anti-obesity potential when consumed [24]. The large numbers of hydroxyl groups on the flavone molecule are responsible for their α-glucosidase, α-amylase, and lipase inhibitory activities, which is the anti-obesity mechanism of their action [24]. 

**Isoflavones** are differentiated from other flavonoids with their planar ring system containing benzenoid B ring attached to carbon number three (C3). Isoflavone-rich foods such as lentils, black soybean, chickpea, peanut, and common beans may demonstrate anti-obesity effects and suppressive ability against oxidation and inflammation associated with obesity [25]. **Anthocyanins** may include and are not limited to delphinidin 3-*O*-rutinoside, malvidin, cyanidin 3-*O*-rutinoside, and cyanidin 3-*O*-glucoside. Food products rich in these compounds, such as wine, berries, and beans, have beneficial effects on obesity [25]. Anthocyanins effectively improve the lipid profile by significantly reducing serum triglyceride and cholesterol levels but increasing high-density lipoprotein-cholesterol concentration in obese conditions [25]. Finally, **flavonols** such as (+)-catechin, (−)-epicatechin, epigallocatechin, and some polymeric procyanidins can be found mostly in fruits, tea, wine and chocolate. They contain a B-ring attached to carbon number two (C2), even though they lack a carbonyl group in their carbon number four (C4) position or double bonds between carbon number two (C2) and number three (C3). Catechin-rich tea suppressed the expression of miR-335 in white adipose tissue [26]. At the same time, proanthocyanin–rich grape seed extracts administered to rats significantly improved obesity parameters [27], proving their efficacy as anti-obesity agents.

### 3.2. Non-Flavonoid-Type Phenolic Compounds in Foods

Non-flavonoid polyphenols are diverse in foods. They are categorized into phenolic acids, lignans, and stilbenes. **Phenolic acids** are widely distributed in foods and can be found in high concentrations in grains, wine and berries. They may include and are not limited to caffeic acid, caftaric acid, ferulic acid, chlorogenic acid, and benzoic acid. Many physicochemical factors such as polarity, plant matrix, digestibility by gastrointestinal enzymes, and molecular mass can affect their bio-accessibility and bioavailability. Foods with high phenolic acid content have beneficial effects against obesity, primarily via modulating gut microbiota composition [22]. On the other hand, **lignans** consist of compounds with 1,4-diarylbutane in their structure. They include pinoresinol, secoisolariciresinol, syringaresinol, matairesinol, and lariciresinol diphenolic. These compounds are mainly found in fruits, vegetables, coffee, tea, and cereal products. When consumed, lignans express anti-obesity activity by inhibiting the expression of adipogenic factors and lipid metabolism-regulating factors during adipocyte differentiation [28]. They are also said to be involved in inducing G0/G1 cell cycle arrest, inhibiting mitotic clonal expansion during the early stage of adipogenesis [28]. The bioavailability of lignans requires biotransformation reactions involving demethylation and dehydroxylation, which are directly dependent on intestinal bacterial metabolism [22]. Moreover, **stilbenoids** may be found in red grapes, cranberries, strawberries, and peanuts [22]. Resveratrol and its derivatives are the most known stilbenoids in the diet and probably the most significant and widely studied stilbenoids. Their metabolized form, such as dihydroresveratrol and 3,4′-dihydroxy-trans-stilbene (from trans-resveratrol), are responsible for their anti-obesity effects.

## 4. Integrating Polyphenols as Bioactive Ingredients in Foods: Foods for Weight Loss

Phenolic compounds are broadly present in the plant kingdom and are arguably the most abundant and essential group of secondary metabolites in plants. Phenols from edible vegetables, cereals, legumes, and fruits are the most consumed dietary polyphenols. Consuming these foods or their products can potentially benefit patients suffering from obesity in disease management. The strategies to control body weight by incorporating active compounds capable of limiting the bioavailability of fats, stimulating energy expenditure, and modifying the composition of the gut microbiota into food products is envisaged as an appropriate method to find alternative treatment for obesity from natural products. There is a growing trend of incorporating bioactive polyphenols with anti-obesity effects into foods to treat and prevent obesity and other diseases [29]. Using food enrichment, encapsulation, and formulation technologies can deliver active ingredients such as polyphenols in processed foods and improve the effectiveness of such foods against obesity [29]. Manipulating products by adding plant bioactive metabolites has become a promising approach for controlling or treating obesity. Such efforts have been successful in producing products such as fruit smoothies with high concentrations of added fruit polyphenols [30], functional bread enriched with fruit polyphenols [31], application of microencapsulation for the safe delivery of green tea polyphenols in foods [32], infant food products such as fruit-based food (from blueberry, cranberry, chokecherry-rich polyphenols) for preventing/treating obesity [33], and drinking yogurt with added fruit polyphenols [33]. In addition to inhibiting obesity development, polyphenol-enriched foods have been stated to prevent obesity-associated disorders. Kiss et al. produced noodles fortified with polyphenol-rich buckwheat and amaranth powders [34]. The antioxidant potential of the fortified product was found to be higher than the control [34]. Mayneris-Perxachs et al. also demonstrated that the supplementation of hesperidin and naringenin in biscuits positively influenced metabolic syndrome in obese rats [35]. They observed reduced body weight, total body fat, total cholesterol, and oxidative stress [35]. Incorporating polyphenols has positive effects in improving the anti-obesity metabolic properties of foods, and consuming such foods has protective effects against the development and progression of obesity. These benefits have been described in Table 2. 

## 5. Molecular Target and Mode of Action for Antiobesity of Polyphenols 

**Figure 3:**
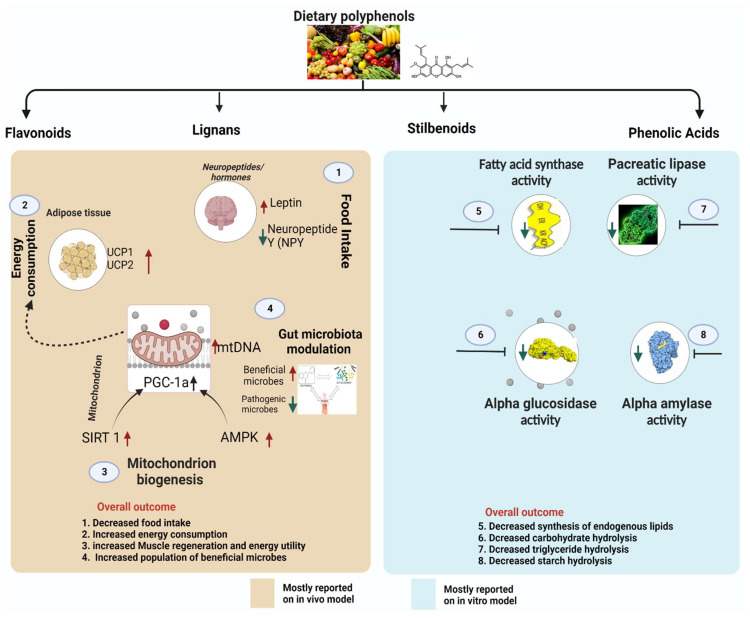
Molecular target and mode of action for antiobesity of polyphenols. **↑,** Up-regulation; **↓,** down-regulation.

The mechanisms through which polyphenols can inhibit obesity include inhibiting digestive enzymes (mainly alpha-glucosidase, pancreatic lipase, fatty acid synthase, and alpha-amylase), stimulating energy expenditure, suppressing appetite, regulating lipid synthesis, and modulation of gut microbiota (Figure 3). These mechanisms may occur individually or in certain combinations, as described below.

### 5.1. Polyphenols Influence Neuropeptides/Hormones Involved in Food Intake and Satiety

There is increasing evidence showing that dietary polyphenols are beneficial in reducing obesity by acting on various neurohormones in the brain involved in food intake and satiety. In vitro and in vivo studies reveal that polyphenols have potential roles in neurohormones that modulate food intake and energy regulation in obesity. Insulin is an important hormone that regulates blood glucose levels in the body; it is adipocytes’ primary hormonal signal for energy storage [43]. The causal links between obesity and insulin are complex and controversial, and research has not been able to establish these links fully. However, insulin hypersecretion by the pancreas has been implicated in obesity development and pathogenesis [43]. Studies have been conducted to assess the relationship between insulin and obesity development. For instance, a study indicated that adults who hypersecreted insulin in response to an intravenous glucose tolerance test showed excessive weight gain over a 15-year follow-up period [44]. Thus, insulin is an essential neurohormone in the pathogenesis of obesity, and therefore, reports on the effects of polyphenols on insulin underscore their potential role in obesity. Long-term intracerebroventricular infusion of resveratrol normalized hyperglycemia and improved hyperinsulinemia in obese mice [45]. Down-regulation of the insulin-like growth factor pathway in medulloblastoma cells was observed upon administration of curcumin [46]. The study suggested that curcumin contains polyphenol metabolites which are influential in the central nervous system’s effects in regulating neurohormones such as insulin [46]. Hormone leptin also plays a crucial role in obesity. Adipose tissue is one of the main organs producing several adipokines, such as leptin regulating energy metabolism. Leptin inhibits neuropeptide Y (NPY), which is orexigenic and stimulates proopiomelanocortin which activates anorexigenic factors inhibiting food intake [47]. The daily consumption of 200 mg/kg of resveratrol restored leptin sensitivity in obese rats and reduced their overall body weight [48]. In addition to their effects on insulin and leptin, polyphenols have been shown to exert anti-obesity by directly modulating neuropeptides in food intake, as anthocyanins were reported to inhibit neuropeptide Y, suppressing obesity in high-fat diet-fed rats [49]. 

### 5.2. Polyphenols Inhibit Pro-Obesity Enzymes

Ingested fats are metabolized and absorbed in the blood through the duodenum. Lipids exist in the body as triglyceride, an ester compound synthesized from a single molecule of glycerol and three fatty acids. Triglycerides cannot be absorbed into the blood in their native form; they must be hydrolyzed into simpler components (monoacylglycerol and free fatty acids) which can easily be absorbed and transported [50]. Human pancreatic lipase is the main enzyme that breaks down dietary fats in the human digestive system [50]. Therefore, an essential target for the treatment of obesity includes the development of pancreatic lipase inhibitors. Polyphenol-rich extracts from a range of plants have been studied for their ability to inhibit pancreatic lipase activity in vitro and in vivo [50]. Buchholz et al. reported that several factors determine the inhibitory effect of polyphenols against pancreatic lipase [50]. First, the potential inhibitory ability depends on the number and position of phenolic hydroxyl groups; polyphenols with a higher number of phenolic hydroxyl groups possess a more significant inhibitory effect against pancreatic lipase [50]. Second, non-esterified polyphenols such as (+)-catechin and (−)-epicatechin have been found to possess a lower inhibitory activity than esterified ones [50]. Third, the degree of polymerization of polyphenols influences their ability to inhibit lipase. The investigation of phenolic acids demonstrated that hydroxybenzoic acids inhibit the activity of lipase less powerfully than hydroxycinnamic acids, probably due to their differences in the degree of polymerization [50]. Finally, Buchholz and Melzig deduced that polyphenols with methoxy groups in the molecule are less potent than those with hydroxyl groups. Polyphenols inhibit pancreatic lipase by binding to the protein moiety [50]. Therefore, the protein-binding affinity of polyphenols is vital in the lipase-inhibitory activity of these compounds [51]; possibly the reason polyphenols with different structures have different lipase-binding affinities. Nonetheless, even though polyphenols may prevent the digestion of lipids by lipase enzymes, it has been reported that as the inhibitory activity of polyphenols proceeds, the lipase enzyme activity would often be compensated by increased secretion of these enzymes in the gut [52]. This phenomenon was observed in a study of condensed tannins in rats [52]. Supplementary Table S1 summarizes research on lipase inhibitory activities of polyphenols and polyphenol-rich extracts.

In most cases, obesity occurs when fat synthesis exceeds fat oxidation. In the body, fat accumulation is tightly maintained through lipogenesis and lipolysis. While lipogenesis converts simple sugars and other substrates to fatty acids and eventually triglycerides, lipolysis hydrolyses triglycerides to generate free fatty acids and mono- or diacylglycerol or free glycerol. The enzyme fatty acid synthase plays a central role in animal de novo lipogenesis. The enzyme catalyzes the conversion of acetyl-CoA and malonyl-CoA to palmitate (a 16-carbon saturated fatty acid). This process is one of the steps involved in the synthesis of endogenous lipids in the body and may lead to the development of obesity if it becomes excessive. Polyphenols can inhibit the enzyme fatty acid synthase activity and block the lipogenic pathway in vivo. Bee bread polyphenols significantly reduced the obesity index by inhibiting fatty acid synthase activity in the high-fat diet-induced obese rats [53]. Epigallocatechin-3-gallate is a potent natural inhibitor of fatty acid synthase and was reported to prevent the enzyme’s activity in prostate cancer cells, thereby reducing endogenous lipid synthesis [54]. Chokeberry-derived polyphenols suppressed fatty acid synthase activity in 3T3-L1 cells, significantly decreasing body weight and serum triglyceride [55]. Additional polyphenols studied for their ability to inhibit fatty acid synthase are summarized in Supplementary Table S2. Screening for fatty acid synthase inhibitors from diverse plant polyphenols could be an important area of research in obesity treatment. Other enzymes that can be significantly involved in obesity development are alpha-amylase and alpha-glucosidase, which hydrolyze starch and carbohydrates, respectively. 

### 5.3. Polyphenols Suppress Lipogenesis of the Adipose Tissue via Obesity-Related Transcription Factors

Obesity is often associated with several disorders, including excessive growth of white adipose tissue [26]. Adipocytes are synthesized from multipotent mesenchymal precursor cells that commit to preadipocytes and then either remain inactive or further differentiate to become mature adipocytes [56]. The process of differentiation is tightly controlled by multiple transcription factors, including miRNA and extracellular hormones such as insulin [56]. The miRNAs are short nucleotides, endogenous non-coding RNAs regulating various biological processes such as adipogenesis and fat metabolism in the body [26]. Of particular relevance, many miRNAs, including miR-335, 103, and 143, are often upregulated during adipogenesis [26,56]. Therefore, substances that can prevent the upregulation of miRNAs may substantially inhibit obesity development. An animal model experiment reported that polyphenols from green tea (especially epigallocatechin gallate) suppressed the expression of miR-335 in white adipose tissue [26]. Since miRNA is a link between weight gain and impaired metabolism in adipose tissue, the study demonstrated that the suppression of miR-335 by polyphenols significantly prevented weight gain and reversed virtually all metabolic complications induced by obesity in mouse white adipose [26]. Besides miRNA inhibition, the beneficial effects of polyphenols in suppressing lipogenesis in adipose tissue have also been linked to their effects on adenosine monophosphate (AMP)-activated protein kinase (AMPK) [57]. AMPK is an important regulator of energy balance in the body and is one of the molecular targets for drugs used to treat obesity [58]. Activation of AMPK protects against diet-induced obesity due to increased body energy expenditure resulting from the high oxygen consumption rate of white adipose tissue (the process is referred to as beiging) [58]. Thus, targeting AMPK activation in adipose tissue can offer a therapeutic strategy for managing obesity. In a study of the anti-obesity activity of green tea polyphenols, Rocha et al. reported a repression of de novo lipogenesis in adipose tissue in diet-induced obese rats, which was accompanied by AMPK activation [57]. Another research showed that gallotannin derivatives from mango suppressed adipogenesis by converting white adipocytes into beige adipocytes in 3T3-L1 adipocytes through the AMPK pathway [59]. Targeting lipogenesis by polyphenols is an effective strategy for managing obesity.

### 5.4. Polyphenols Modulate Thermogenesis and Mitochondrial Biogenesis

Mitochondria functions to produce energy via oxidative phosphorylation and are also involved in various cellular roles in the body, including apoptosis, calcium balance, and the production of free radicles [60]. Due to these multiple functions, mitochondrial dysfunction can directly or indirectly trigger the origin of numerous diseases, such as obesity and diabetes [60]. Previous studies have demonstrated that obesity development can be linked to decreased mitochondrial respiration, mitophagy signaling, increased production of free radicles, and apoptosis [61]. Therefore, therapeutic strategies directed toward restoring mitochondrial function are promising methods in the treatment of obesity. Peroxisome proliferator-activated receptor-gamma coactivator 1 (PGC-1) consisting of family members, PGC-1α, PGC-1β, and PRC (PGC-1-related coactivator) is one of the key regulators of mitochondrial functions [62]. The PGC-1α coactivators, for instance, regulate the expression of mitochondrial transcription factor A (TFAM), which controls mitochondrial DNA replication and transcription [62]. These activities are managed through phosphorylation, methylation, and acetylation processes. Reversible acetylation of PGC-1α regulated by sirtuin 1 (SIRT1) has been shown to substantially alter mitochondria’s transcriptional activity, leading to various health problems, and excessive weight gain is one of them [60,62].

Recently, it has been shown that markers of mitochondrial biogenesis are upregulated in polyphenol-treated mice (Supplementary Figure S1). An in vivo study also revealed that resveratrol, increasing SIRT1 activity, modulated PGC-1a functions and impacted the regulation of energy balance [63]. The study also showed that resveratrol potently induced mitochondrial activity by not only activating PGC-1a but also increasing oxidative Type-I muscle fibers [63]. These effects triggered by resveratrol were crucial in enhancing the animals’ resistance to diet-induced obesity and tolerating insulin resistance [63]. On the other hand, thermogenesis is a process in which heat is produced in the body, mainly due to basal metabolism. Most animals regulate thermal homeostasis via brown adipose tissue. Thus, brown adipose tissue has been identified as a critical site for energy expenditure in the form of thermogenesis. In low temperatures, sympathetic nerves are stimulated to activate brown adipose tissue via β3-adrenoreceptors to facilitate thermoregulation [60]. Additionally, thermogenesis in brown adipose tissue can be stimulated by diet-induced stress, which is crucial in obesity [60]. Numerous reports have investigated how thermogenesis regulation via brown adipose tissue plays a protective role against obesity [64,65] and the potential effects of polyphenols in this process [66,67]. Vanillic acid reduces body weight gain and maintains body temperature by promoting thermogenesis and mitochondrial biogenesis of brown adipose tissue [66]. According to the report, vanillic acid-activated brown adipose tissue thermogenesis promoted inguinal white adipose tissue browning, thereby inhibiting obesity development [66]. Epigallocatechin-3-gallate feeding of mice decreased body weight gain and plasma and liver lipids [67]. According to the study, the treated mice exhibited higher body temperature and increased mtDNA content in brown adipose tissue, indicating increased thermogenesis and mitochondrial biogenesis [67]. Therefore, the research on the effects of polyphenols on thermogenesis and mitochondrial biogenesis may enhance understanding of the importance of these compounds in the body and provide hope for developing functional foods to prevent or treat obesity. Supplementary Table S3 summarizes studies on the roles of polyphenols in inducing thermogenesis and mitochondrial biogenesis.

### 5.5. Gut Microbiota Modulation 

Human gut microbiota (GM) is a complex ecosystem. Gut microbiota performs critical roles in diet processing, eventually influencing several physiological functions in the host organism. These functions include harvesting energy from indigestible food, altering fatty acid oxidation, controlling satiety, lipogenesis, production of bile acid, and affecting innate immunity [68]. Thus, gut microbiota dysbiosis can cause the progression of various chronic conditions related to these activities. Diet represents an essential factor in regulating the symbiotic relationship of mammalian gut microbiota since it provides the energy and substrates for the life and growth of the organisms. The interaction between dietary polyphenols and gut microbiota has been well documented [68]. There is a two-way interaction between polyphenols and the gut microbe that affect human metabolism and could reduce the risk of obesity: gut microbiota enzymatically biotransforms polyphenol structure producing the metabolized forms of the compounds (discussed in Section 5), while polyphenols can potentially change gut microbiota composition by inhibiting the growth of pathogenic bacteria but enhancing the growth of beneficial microbes. Thus, gut microbiota can regulate the bioavailability of the unabsorbed polyphenols, which reciprocally modulate their functions [69].

The mechanisms through which phenolic compounds modulate the gut microbiota is a subject that requires further elucidation. The heterogeneity of the polyphenols, their food sources, their coexistence with other bioactive compounds within a regular diet, and the complexity of the human gut microbiome make it challenging to understand how polyphenols modulate the microbial composition of the human gut. However, the mechanisms may involve both direct and indirect interactions. Phenolic compounds can simultaneously stimulate beneficial bacteria growth but inhibit pathogenic bacterial proliferation through their bactericidal or bacteriostatic effects in the gut [70]. The imbalance between *Firmicutes* and *Bacteriodetes* has been associated with obesity development and insulin resistance [70]. It has been found that a decreased ratio of *Firmicutes/Bacteroidetes* (F/B) can help prevent the development and progression of obesity [69]. In a study of catechin-rich tea infusion, a significant decrease in *Firmicutes* and increases in the number of *Bacteroidetes* and *Proteobacteria* were observed, which correlated to overall reduced body weight in a mouse model of high-fat-diet-induced obesity [69]. The role of foods in enhancing the beneficial microbes and reducing pathogenic ones at a species level is of particular importance. Feeding C57BL/6J mice with a high-fat diet supplemented with three types of tea (green tea, oolong tea, and black tea) infusions for 13 weeks was found to increase diversity and positive change in the microbial community in the gut, which consequently decreased the accumulation of adipose tissue in the mice [71]. This study revealed that phenolic acids, flavonols, and alkaloids from the three types of tea modulated the composition of gut microbiota such that *Alistipes*, *Rikenella*, and *Akkermansia* were increased, thereby preventing obesity development [71]. Additionally, one of the key species of beneficial bacteria in the gut is the mucin-degrading species of bacteria, *Akkermansia muciniphila.* Recently, *Akkermansia muciniphila* has received increasing attention because of its ability to improve body weight and protect against various features of metabolic syndrome [72]. Polyphenol-rich cranberry extract administration was found to revert an essential shift in the gut microbiota of mice by triggering an increase in the relative abundance of *Akkermansia* in high-fat/high-sucrose-fed rats [72]. Thus, plant foods rich in polyphenols can modulate microbiota composition, forming a baseline that might offer a dietary intervention strategy for obesity treatment. Supplementary Table S4 summarizes the studies on the effects of polyphenols on gut microbiota composition in obesity.

## 6. A Comprehensive Review of Cellular, Animal, and Human Models Investigating the Potential of Polyphenol-Rich Extracts against Obesity

### 6.1. Studies on the Anti-Obesity Effects of Polyphenol-Rich Extracts Performed in Cell and Animal Models

*Studies on cell model*: After in vitro, ex vivo studies are the second step in establishing the effects of plant phytochemicals on health. Plant extracts’ in vitro anti-obesity potential is often assessed by evaluating their inhibitory activities against pancreatic lipase, alpha-glucosidase, alpha-amylase, and fatty acid synthase enzymes (discussed above). However, to understand the initial molecular mechanism undergoing obesity in ex vivo/in vitro, the cell culture model (mostly, 3T3-L1 cell line) has been used as one of the most reliable models for evaluating the cell differentiation of preadipocytes into adipocytes. Accumulation of fat and the differentiation of adipocytes are often related to obesity development. Soeng et al. found that rambutan seed extract (rich in polyphenols) decreased triglyceride levels and inhibited glucose-6-phosphate dehydrogenase (G6PDH), which promotes adipogenesis, thereby reducing obesity in ex vivo in the 3T3-L1 cell line [73]. Treatment with black soybean anthocyanins at a concentration of 12.5–50 μg/mL prevented obesity development by inhibiting the proliferation of both preconfluent preadipocytes and mature postconfluent adipocytes in 3T3-L1 cells [74]. Pinent and colleagues discovered that grape-seed-derived procyanidins treatment inhibited adipogenesis in the 3T3-L1 cell, mainly at the induction of differentiation [75]. Chokeberry polyphenols suppressed fatty acid synthase activity in 3T3-L1 cells [55]. The study found that by inhibiting the enzymes, polyphenol-rich Chokeberry significantly decreased the animal body weight and serum triglyceride [55].

*Studies on animal models:* The anti-obesity potential of polyphenols-rich plant extracts have not been demonstrated in vitro and ex vivo studies alone; their anti-obesity properties have also been shown in animal models as in vivo evidence. Even though the anti-obesity mechanisms of action depend on the extract and the animal model used, studies agree on general anti-obesity events that occur with polyphenol intake. Most studies report the reduction of animal body weight, fat tissue size, and downregulation of pro-obesity markers when animals such as mice are fed a mixture of polyphenol-rich extract and a high-fat diet. Furthermore, there is agreement that the intake of polyphenol-rich extracts improves serum triglycerides and reduces total cholesterol and LDL-cholesterol in plasma. Cellular and animal studies on the potential effects of polyphenol-rich extracts on obesity were summarized in Table 3.

### 6.2. Studies on the Anti-Obesity Effect of Polyphenol-Rich Plant Extracts Performed in Humans

The impact of polyphenols supplementation should be tested on human subjects before consumption. The US Food and Drug Administration only considers human clinical trials as strong evidence about a health claim of a bioactive compound. Despite the increased research on polyphenols as anti-obesity agents, only a few clinical trials have effectively evaluated the efficacy of these compounds on obesity. A number of polyphenol-rich plant extracts have been investigated for their ability to reduce obesity development in human clinical trials. Grape, orange, and citrus fruits are increasingly being investigated for their anti-obesity effects. Despite most studies being carried out in vitro and in vivo, some clinical trials have reported grape, orange, and citrus fruits as anti-obesity effects. Dallas et al. described a significant improvement in obesity parameters when a commercial drink consisting of a mixture of polyphenol-rich citrus extract, orange, grapefruit, sweet orange, and guarana was investigated for their anti-obesity effects [83]. The 95 obese participants who consumed two capsules of citrus polyphenol extract containing orange, grapefruit, sweet orange, and guarana for 12 weeks had reduced body weight and abdominal fat [83]. The summary of the anti-obesity effects of polyphenol-rich extracts has been summarized in Table 4.

### 6.3. Anti-Obesity Effects of Commonly Studied Polyphenols-Rich Foods 

**Figure 4:**
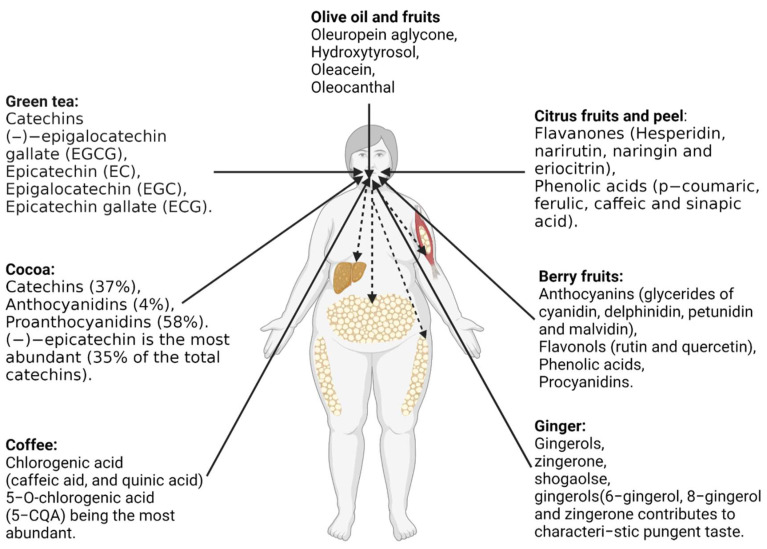
Major Polyphenols responsible for anti-obesity effects in green tea, cocoa, coffee, berry, citrus fruits, and ginger.

Recently, research has highlighted the bioactive roles of polyphenols against obesity. Numerous foods, consisting of grains, fruits and vegetables, have been studied for their anti-obesity effects. However, current research has paid particular attention to food materials known to contain a certain amount of polyphenols, enough to exert anti-obesogenic effects when consumed. The attention has been put particularly on green tea, berry fruits, citrus fruits, coffee, cocoa, and ginger polyphenols, with an effort to advance their extraction, characterization, and understanding of their health benefits (Figure 4).

*Green tea polyphenols:* Green tea is one of the most popular worldwide beverages. Green tea extract contains diverse polyphenolic contents and is particularly abundant in catechins with various health effects. Polyphenol-rich green tea extract is one of the most studied for its health activities. Catechins are the most abundant polyphenols with known biological activities in tea. Catechins are present from 15%–20% by weight in green tea. Epigallocatechin gallate (EGCG), a kind of catechin, exerts an inhibitory effect on acetyl-CoA carboxylase, thereby preventing obesity development [91]. Polyphenol-rich green tea extracts have been widely studied for their anti-obesity effects. The potential mechanism of how green tea catechin induces anti-obesity effects involves changes in fatty acid oxidation and metabolism. For example, under the influence of the sympathetic nerve, norepinephrine (NE) stimulates lipolysis in peripheral tissues, including adipose, liver, and skeletal muscle, releasing free fatty acids into circulation while at the same time up-regulating hepatic lipid metabolism [92]. Studies in rodents have shown that green tea polyphenols could prevent obesity by stimulating lipolysis in peripheral tissues (adipose, liver, and skeletal muscle), releasing a free hypothesis [93,94]. Randomized, controlled intervention trials have also confirmed that consuming tea rich in polyphenols exerts beneficial effects against obesity via these mechanisms [95]. However, the anti-obesity effects of green tea polyphenols is described as a cumulative process that occurs over time in humans [92]. Auvichayapat et al. evaluated the effects of green tea catechin (GTC) ingestion (141 mg GTC + 87 mg caffeine) as part of a weight loss program among Thai men and women [96]. During the 8th and 12th weeks of the study period, Auvichayapat et al. reported that loss in body weight was significantly increased by supplementation. A 183.38 kJ/day difference in resting energy expenditure was observed, while the difference was 0.02 for the respiratory quotient. Thus, the researchers concluded that green tea could reduce body weight by enhancing energy expenditure and increasing fat oxidation [96].

*Berry fruit polyphenols*: In recent years, demand for fresh berry fruits and berry products has dramatically increased, which largely fuels the cultivation and production of berry fruits. Raspberries, blueberries, mulberries, lingonberries, blackberries, black chokeberries, elderberries, cranberries, and strawberries are vast reservoirs of polyphenols and other bioactive metabolites. Their anti-obesity potentials remain significantly attractive. Jiang and colleagues comprehensively reviewed the effects of berry fruits as anti-obesity foods [97]. Polyphenols, including anthocyanins (mainly glycerides of cyanidin, delphinidin, petunidin and malvidin), flavonols (rutin and quercetin), phenolic acids, and procyanidins have been identified in berries [97]. From the perspective of actual clinical experiments, the anti-obesity of freeze-dried blueberry powder was performed on 32 men and women with BMI between 32 and 45 kg/m^2^ [98]. The study subjects consumed 22.5 g of blueberry powder twice daily for 6 weeks; the supplement contained 32.49 mg/g of total phenolics; 14.84 mg/g of anthocyanins. The supplementation improved body weight and insulin sensitivity at the end of the study period [98]. A mixture of cranberry and strawberry polyphenol extracts was evaluated for the ability to reduce obesity among 116 subjects (BMI ≥ 25 kg/m^2^) [99]. The extracts contained 20.04 mg/120 mL of proanthocyanidins and 28.206 mg/120 mL of phenolic acids, and the subject consumed 120 mL of beverage daily for 6 weeks. The supplementation improved insulin sensitivity and other obesity parameters [99]. Moreover, 38 subjects (22 women and 16 men) consumed 45 g lyophilized berries 100 mg extract thrice daily for 2 months [100]. The supplement consisted of 60 mg/100 mg of total polyphenols, with 20 mg/100 mg of anthocyanins (cyanidin 3-galactoside (64.5%) and cyanidin 3-arabinoside (28.9%). The supplement was able to restore to improve tested parameters [100]. The anti-obesity mechanism of berry fruits may be due to the regulation of lipid metabolism, including suppressing lipogenesis and improving fatty acid oxidation. Additionally, the ingestion of berry fruits such as mulberry has also been reported to prevent obesity by enhancing the growth of beneficial gut microbes [101]. 

*Citrus polyphenols:* Polyphenols from citrus have been evaluated as one of the attempts to prevent and treat obesity. Experimental results are not entirely consistent; however, most of the published papers attribute the anti-obesity effects of citrus polyphenols to their impact in reducing adipose tissue, increasing biochemical reactions related to fat oxidation as well as improving the serum lipid profile. Citrus fruits, consisting mainly of flavonoids as the primary polyphenols, are the most studied citrus products [102]. Among the flavonoids in citrus are flavanones, flavones, flavonols, and anthocyanins. Hesperidin, narirutin, naringin, and eriocitrin are the most known flavanones in citrus fruits [102]. Other phenolic compounds, including p-coumaric, ferulic, caffeic and sinapic acids, can also be found in these foods in significant amounts. Although not often studied for their health effects, bitter orange is a good source of flavonoids that could exert anti-obesity effects. Eight-week-administration of bitter orange (*Citrus aurantium Linné*) in high-fat diet-induced obese mice resulted in a significant decrease in body weight, adipose tissue weight and serum cholesterol [103]. Moreover, in further in vitro study, sinetrol (citrus-based polyphenolic dietary supplement) inhibited cAMP-phosphodiesterase in cell models and human clinical studies [104], whereas lemon peel polyphenols enhanced peroxisomal β-oxidation through up-regulation of mRNA levels of PPAR α and acyl-CoA oxidase in in-vivo mice model study [105]. Thus, regulation of lipid metabolism, energy expenditure and adipogenesis has been described as significant mechanisms of anti-obesity effects by citrus polyphenol extracts.

*Coffee and cocoa polyphenols:* Cocoa comprises over 380 known bioactive components, 10 of which are considered psychoactive compounds. In their original form, cocoa beans are inedible due to their high concentration of polyphenols, which often contribute to the unfavorable bitter flavor [106]. However, processed cocoa products, such as chocolate, contain reduced total polyphenolic content, a decrease from 100% to about 10% in levels [106]. Three groups of polyphenols, namely catechins, anthocyanidins, and proanthocyanidins, are known to be the dominant compounds in cocoa. Of the total polyphenols, catechins constitute 37%, anthocyanidins comprise 4%, and proanthocyanidins about 58%. (−)-epicatechin is the most abundant, constituting 35% of the total catechins in cocoa beans, while (+)-catechin, (+)-gallocatechin, and (−)-epigallocatechin are only present in trace amounts [107]. Cocoa beans and cocoa-based products have been consumed over decades. Attempts have been made to evaluate the anti-obesity effect of cocoa beans and other cocoa-derived products in the past years. Golomb et al. conducted a cross-sectional study to investigate the impact of chocolate (cocoa-based product) intake on the body weight of 1018 subjects [85]. The researchers noted that chocolate consumption decreased the subjects’ BMI and overall body weight [85]. Ferrazzano et al. [108] discovered that mice fed with a cocoa-enriched diet had reduced body weight, mostly due to decreased adipose tissue synthesis. The researchers concluded that the polyphenols contained in cocoa might have significantly contributed to anti-obesity effects by decreasing fat synthesis. Cocoa and chocolate consumption may also have beneficial effects on satiety, which may help prevent weight gain [107]. 

On the other hand, coffee is the second most consumed beverage in the world, after tea. Its health benefits have been largely associated with its main component, caffeine. However, coffee contains many other bioactive compounds, approximately 2000 different chemicals. The primary polyphenols in coffee are chlorogenic acid and its derivatives, which account for 3% *w*/*w* of the roasted coffee powder. A single cup of coffee is estimated to contain 20–675 mg of chlorogenic acid. The anti-obesity effect of coffee has been studied, especially for at least 10 years. The findings have shown that coffee polyphenols are effective anti-obesity agents. For example, 44 patients with non-alcoholic fatty liver disease were enrolled in a double-blind, placebo-controlled clinical trial and then administered green coffee bean extract (1 g/day) for 8 weeks [109]. Supplementation with green coffee bean extract improved the levels of triglyceride, total cholesterol, free fatty acids, and fasting blood sugar [109]. A study conducted among 93,179 individuals showed that coffee intake of up to four cups/day lowered the risk of obesity with an odds ratio (ORs) of 0.82–0.86, compared with non-coffee drinkers [110]; however, this study failed to specify metabolites which were responsible for the observed effects.

*Ginger polyphenols:* Ginger (*Zingiber officinale*) is a popular spice and vegetable used as a traditional medicine in many countries to treat various diseases. It has three primary phenolic compounds, namely: gingerols, zingerone, and shogaols. Gingerols contribute to the pungent taste of ginger. The beneficial effect of ginger on obesity prevention and treatment has been recently considered, and some promising results from experimental animals have been published [111,112]. It is reported that polyphenols in ginger could influence many vital parameters of obesity through mechanisms such as stimulating enhanced thermogenesis and energy expenditure, suppressing appetite, stimulating lipolysis, and inhibiting intestinal absorption of dietary fat [112,113]. However, most of these results are preliminaries since they have yet to be confirmed in clinical trials. Ginger extracts suppressed the expression of genes related to adipogenesistor γ (PPAR-γ) and adipocytes in an animal model [112]. When obese rats were fed with ginger extracts, serum metabolites were significantly restored, and glucose tolerance was improved [112]. In a clinical trial, a randomized, double-blind, placebo-controlled was performed using steamed ginger ethanolic extract (SGE) containing a high 6-shogaol content [114]. Following the supplementation period, mean body weight, body mass index, and body fat level were significantly lower in the SGE group than in the placebo group [114], confirming polyphenolrich ginger extract as anti-obesity agents. 

*Olive polyphenols*: Recently, among the most known polyphenols sources are olive products. The high polyphenol content in these products has aroused growing interest, and studies have been carried out to determine their potential therapeutic ability. For olive oil, the most studied olive product, the major polyphenols associated with its health benefits include oleuropein aglycone, hydroxytyrosol, oleacein, and oleocanthal [115]. Therefore, the health functions and sensory attributes of olive oil depend not only on the content of free fatty acids but also on its levels of polyphenols. Indeed, polyphenols are responsible for the taste of olives. Nonetheless, factors such as cultivar, environmental conditions, cultivation practices, and fruit ripening stage affect the levels of polyphenols in olive oil [115]. Polyphenols derived from olive oil have been reported to modulate obesity. A clinical study involving the intake of 330 mL of olive oil leaf tea 3 times daily during mealtime for 12 weeks demonstrated the efficacy of olive leaf against obesity development [116]. After the intervention, serum triglycerides and low-density lipoprotein cholesterol levels decreased significantly in the oil-leaf tea-treated group (*n* = 28). Thus, olive products have been found to have lipid-lowering effects.

## 7. Bioavailability of Polyphenols, Metabolism by Gut Microbes, Post Absorption Fate, and Eventual Effect on Obesity 

For polyphenols to exert health benefits, they must be absorbed by the human body. Most polyphenols are absorbed through an active transport mechanism that depends on the presence of sodium-dependent glucose transporter 1 (SLGT1), a protein entrenched along the epithelium cell walls [117]. Once absorbed, polyphenols and their metabolites reach tissues and influence the activities of a target tissue/organs related to a disease, thereby improving health. However, the ability of phenolic compounds to be absorbed into the body depends on their bioavailability, which is affected by several factors. The bioavailability of polyphenols can be influenced by the presence of other food components, such as lipids, carbohydrates and proteins when consumed simultaneously [117,118]. For instance, a clinical trial in humans demonstrated that the bioavailability of chlorogenic acids derived from coffee could be reduced by a matrix consisting of coffee and milk (when coffee and milk are consumed simultaneously [119]. In contrast, some food matrices, such as natural almond skin, can enhance the accessibility of polyphenols, while polyphenol-rich cinnamon extract can be incorporated into defatted soy flour containing high protein to improve polyphenol bioavailability [118]. In addition to the effects from the surrounding food components, the hydrophobicity of polyphenols can also significantly affect their absorption along the gut. Hydrophobic polyphenols have relatively low solubility in water, gastric fluids, and small intestine fluids; they often precipitate in these fluids, reducing the amount available for absorption [117]. Moreover, the presence of gut microbial enzymes that hydrolyze phenolic compounds into more minor metabolites significantly affects their bioavailability [117]. 

Polyphenol metabolism along the gut involves a series of stages. It is believed that phenolic compounds derived from diets are frequently conjugated as glycosides, which change to aglycones when metabolized by gut microbiota [68]. The gut microbiota hydrolyses these polyphenol glycosides and esters, reduces their nonaromatic alkenes, and cleaves their overall skeletons [68]. The result is the generation of less complex products, such as phenolic acids and hydroxycinnamates. Only 5–10% of polyphenols can be directly absorbed in the small intestine, while the remaining (90–95%) reach the colon, where microbial enzymes degrade them before their absorption [68,69]. Some of the bacteria related to the degradation of polyphenols include *Lactobacillus spp., Enterococcus casseliflavus, Flavonifractor plautii, Slackia equolifaciens, Eubacterium ramulus, Eggerthella lenta*, and *Bifidobacterium spp.* [25]. *Eubacterium* metabolizes flavonoids, while *Bifidobacterium* and *Lactobacillus* species are involved in releasing hydroxycinnamic acids from the parent compound in the colon [25]. Meanwhile, *Bacteroidetes* and *Firmicutes* are the major groups involved in the colonic metabolism of undigested food remnants, including unabsorbed polyphenols [69]. The gut microbiota contains large quantities of various enzymes that modify food components before they are used in the body or released as waste. Enzymes such as glycosidases, amidases, and esterases catalyze various reactions, including decarboxylation, oxidation, reduction, demethylation, isomerization, and ring cleavage, leading to the production of several types of catabolites of dietary polyphenols [120]. In the mouth, the mastication process takes place; polyphenols interact with digestive enzymes, which destroy their structure as digestion begins. In the stomach, the compounds are released from the food matrix, and the polymeric polyphenols and their glycosidic bond are hydrolyzed by acids [121]. However, most of the polyphenol glycosides resist acid hydrolysis in the stomach and reach the small intestine intact. Once they arrive in the small intestine, enzymatic deglycosylation of polyphenols and absorption of about 5–10% polyphenols take place [121]. 

**Figure 5:**
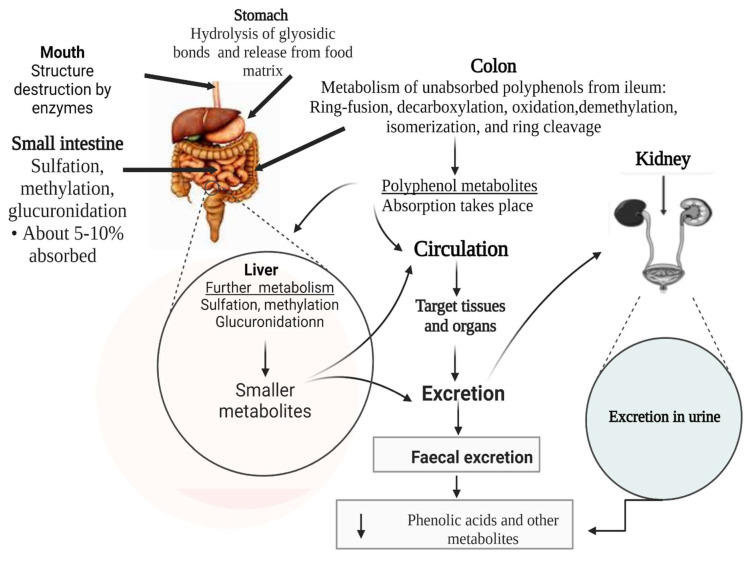
Metabolism fate of polyphenols by the human gut microbiota. In the mouth, the structure of polyphenols is broken down by enzymes, releasing the compounds from the food matrix. In the stomach, the polymeric polyphenols and their glycosidic bonds are hydrolyzed as the digestion process continues. Once they reach the small intestine, polyphenols undergo enzymatic deglycosylation, and absorption of some digested compounds (5–10%) takes place. The absorbed phenolic compounds enter the circulation and reach the liver, where they are metabolized further into smaller metabolites. At the same time, in the large intestine, gut microbiota hydrolyses unabsorbed polyphenols, making them available for absorption into the blood stream. Eventually, polyphenols metabolites are released from the body either through fecal matter or urine excretion.

Absorbed polyphenol products, through the bloodstream, rapidly reach the liver, where they are further hydrolyzed by phase II metabolism before they are eventually excreted outside the body via urine. At the same time, the unabsorbed polyphenols, mostly flavonoids linked to a rhamnose moiety, organic acids, lipids, and polymers, and those bound to dietary fiber and protein, as well as hydroxycinnamic acids esterified to sugars, reach the colon for further digestion; they cannot be directly absorbed in the small intestine. In the colon, gut microbiota hydrolyses the unabsorbed polyphenols to give rise to small phenolic acids and aromatic catabolites [120,121]. These polyphenol by-products are absorbed into the blood and arrive in the liver, where they are further metabolized before being released in urine; however, some of the unabsorbed metabolites from the parent polyphenols are eliminated via feces [120,121]. Figure 5 offers a detailed summary of the polyphenol metabolic process along the human gut. 

There is a huge body of literature evidence reporting on the biological functions of polyphenol metabolites generated by gut microbiota-mediated biotransformation [25]. The microbial enzymes may eliminate glycosides, glucuronides, and sulfates from unabsorbed polyphenols producing aglycons, which are further digested into ring-fission products depending on the type of polyphenol involved. Flavonols such as quercetin-3-O-glucoside are hydrolyzed into their metabolite-derivative products by gut microbiota at their A and B rings. Enterococcus casseliflavus hydrolyses sugar moieties of quercetin-3-*O*-glucoside to release the aglycone quercetin and products such as lactate, acetate, and ethanol [25]. In contrast, Eubacterium ramulus and Clostridium strains metabolize quercetin, forming short-chain fatty acids (acetate, propionate, and butyrate) and other products [25]. Short-chain fatty acids are crucial metabolites in reducing obesity. The anti-obesity benefits associated with short-chain fatty acids have been reported (Table 5). 

The bacteria, *Clostridium coccoides*, *Bifidobacterium* spp., *Eggerthella lenta*, *Adlercreutzia equolifaciens*, *Slackia equolifaciens*, and *Flavonifractor plautii* through hydrolysis of ester bonds, carbon-ring cleavage, or dihydroxylation metabolizes **flavonols** and **proanthocyanidins** into various forms of phenolic acids [25]. The degradation pathway of **flavanones** is similar to flavonols. The first step in the hydrolysis of naringin, a flavanone, involves a deglycosylation reaction which produces a naringenin [22]. The naringenin is biotransformed into phloroglucinol and 3-(3,4-dihydroxyphenyl) propionic acid via the cleavage of the C-ring [22]. The Isoflavone group is biotransformed into their aglycones by β-glucosidase released by gut microbiota. The aglycones can either be absorbed completely or further hydrolyzed into their metabolite forms; for example, daidzein can be converted to *O*-demethylangolensin (*O*-DMA) and equol; genistein into *p*-ethylphenol and 4-hydroxyphenyl-2-propionic acid [22]. **Flavanols** are hydrolyzed into several *O*-sulfated, *O*-glucuronidated, and *O*-methylated forms by gut microbiota. Epigallocatechin gallate, a flavanol, is biotransformed by *Eubacterium* sp. strain into 1-(3′,5′-dihydroxyphenyl)-3-(2″,4″,6″-trihydroxyphenyl)propan-2-ol [127]. It is reported that **phenolic acids** such as ferulic acid can be bound to each other through linkages (8-*O*-4- or 5–5-linkages), forming dimers [22]. The gut microbes hydrolyze the 8-*O*-4 into monomeric ferulic acid, which is eventually biotransformed into 3-(3′,4′-dihydroxyphenyl) propionic acid, 3′,4′-dihydroxyphenyl acetic acid, 3-phenylpropionic acid, and benzoic acid [22]. Finally, as already been mentioned earlier in this article, **stilbenes** such as resveratrol trans-resveratrol are metabolized by gut microbiota, forming dihydroresveratrol, 3,4′-dihydroxy-trans-stilbene, and 3,4′-dihydroxybibenzyl. 

**Figure 6:**
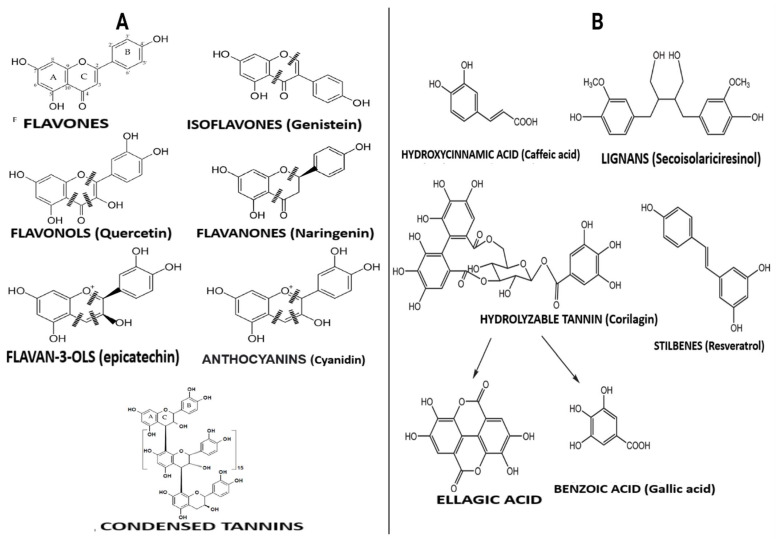
Flavonoids and nonflavonoid-type phenolic compounds that are metabolized by the gut bacteria. (**A**) C-ring cleavage of flavonoids. (**B**) Nonflavonoid-type phenolic compounds metabolized by the gut bacteria. Reproduced from Ozdal et al. [22]. Last accessed on 2 July 2022.

The smaller forms of polyphenols are believed to reflect precisely the physiological effects of their parent compounds [120]. The microbiota-gut-brain axis is considered a neuroendocrine system that plays a vital role in activities controlled by the central nervous system [128]. In addition to modulating gut bacterial composition, the polyphenol metabolites can modulate brain biochemistry or directly act as neurotransmitters through the microbiota-gut-brain axis, thereby affecting body physiological activities of the brain, including those related to stress response, appetite, inflammatory injury, and obesity [128]. Figure 6 is a description of various structures of polyphenols and their metabolic products.

In their metabolized forms, the bioavailability of polyphenols is improved, and they can effectively reach the target site of the disease and exert health benefits. In fact, the anti-obesity ability of most polyphenols is based on the properties of their end product of metabolism. Thus, most of the products of polyphenol metabolism by gut microbial have been reported to possess anti-obesity effects. Products from epicatechin metabolism such as 1,3,5-Trimethoxybenzene have been shown to inhibit adipocyte differentiation, while dihydroxyphenylpropionic acid and 3,4-dihydroxybenzoic acid, metabolic products of flavan-3-ols possess pancreatic inhibitory effects (see Table 6).

## 8. Improving the Bioavailability and Delivery of Polyphenols in the Body 

The bioavailability of most phenolic compounds is closely related to their chemical structure. Most polyphenols exist as esters, glycosides or polymers, which are not readily available in the body in this native form. Gut intestinal transformations of polyphenols produce bioavailable and active phenolic metabolites that can easily be absorbed into the body. In their metabolized condition, these compounds can reach tissues and the brain, where they exert various biological effects. However, it is essential to emphasize that the low bioavailability of polyphenols is one of the critical drawbacks in their utilization as functional ingredients to improve health. It is recognized that microbes in the gut are the main metabolizers of insoluble and unabsorbed polyphenols. However, it is also perceived that gut bacteria may not freely circulate along the gastrointestinal tract (GIT) without encountering harsh environments that may kill them [147]. Hence, the degradation of polyphenols may not be entirely possible in all regions of the GIT, further complicating their bioavailability. Thus, to overcome this challenge, there have been proposals to safely deliver readily-available plant active ingredients into the body using methods exploiting physiological changes in the GIT, such as osmotic control, to improve the functions of these compounds [147]. 

**Figure 7:**
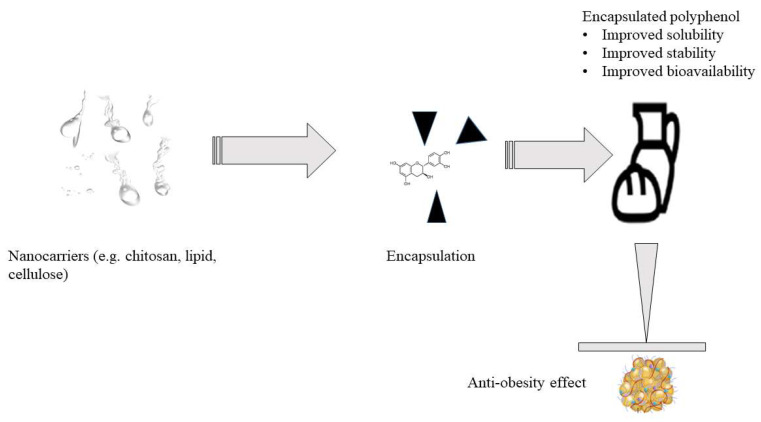
Proposed encapsulation process of polyphenols.

Overall different strategies can be employed to enhance the bioavailability of bioactive compounds: (i) nano-delivery systems, which demonstrate potential for the protection of these compounds during food processing or digestion process; (ii) absorption enhancer materials which facilitate bioactive compounds’ membrane permeation; and (iii) excipient foods with the ability to improve nutraceuticals’ biological activity [148]. Several micro- and nano-encapsulation systems have been proposed to deliver individual polyphenols or their mixtures to enhance their solubility, instability, and poor permeability in the body. Nevertheless, overall knowledge of this technology needs to be improved. The properties of micro- and nanoparticles, including their shape, surface, and stability, are thought to strongly affect active compounds’ in vitro and in vivo fate to improve functionality [147]. In the literature, polyphenols-microencapsulation has been applied to enhance the bioavailability of polyphenolic compounds (Supplementary Table S5). Flavonoids are anti-obesity agents with therapeutic ability based on oral absorption and the properties of their end product of metabolism. However, most flavonoids have a low dissolution rate from solid oral forms and cleavage in the gut lumen. Thus, a diverse range of cellulose derivatives with gastro-resistant swelling effects and controlled release properties, such as cross-linked carboxymethylcellulose and cellulose acetate trimellitate, have been used to improve their functionality to deliver flavonoids in vivo and in vitro extract [149]. Microencapsulation technology has also been used in processed foods to deliver cocoa hull polyphenols into bakery products [150] and green tea polyphenols in functional bread [32]. Figure 7 describes the proposed encapsulation process of polyphenols.

Recent developments in the design of bio-based delivery systems have also led to the use of emulsions, liposomes, and hydrogels to improve the delivery and bioavailability of polyphenols in the body. Emulsions are thermodynamically unstable colloidal dispersions typically assembled from two or more immiscible fluids. Nowadays, emulsions can be utilized to encapsulate polyphenols to enhance their bioavailability; oil-in-water is the most commonly used emulsion to encapsulate polyphenols such as quercetin [117]. Quercetin has been incorporated into perilla oil-in-water emulsions as a black bean protein-quercetin complex that acts as an emulsifier [117]. Polyphenols such as curcumin, resveratrol, and quercetin were loaded on different nanoemulsion-based delivery systems to improve the efficacy of their lipophilic nature [151]. On the other hand, liposomes have also been used as delivery systems for polyphenols; they can protect polyphenols against hydrolysis by enzymes and acids in the stomach; thus, polyphenols can reach their target tissues [117]. The interiors of hydrogels contain a porous three-dimensional polymer network that can hold about 90% of water; thus, hydrogels have been used to enhance the stability and bioavailability of polyphenols. A study showed that injectable hydrogels based on curcumin could improve the effectiveness of this polyphenol against cancer cells [152]. In general, modern encapsulation systems are currently leading to a new era of developing diet-based medicine. As the demand for polyphenol-based nutritional supplements and edibles grows, the utilization of encapsulation systems also grows.

## 9. Effects of Processing on Polyphenol Content of Foods and Ultimate Impact on Anti-Obesity Potential of the Final Product

Food processing can influence the metabolites in foods (their content and bioavailability). Effects of traditional technologies such as thermal processing and fermentation on the polyphenols content of food have been evaluated, and the eventual impact on the anti-obesity potential of the final products has been established [153,154,155]. In the literature, fermentation of tea leaves using *L. paracasei* subsp. *paracasei* NTU 101 was able to enhance the content of polyphenols such as EGC (107.9 versus 159.6 μg/mL), EGCG (482.4 versus 968.3 μg/mL), and chlorogenic acid (38.5 versus 46.9 μg/mL) compared to the unfermented [153]. The final product of the fermented tea leaves showed better anti-obesity efficacy compared to the unfermented tea [153]. Thermal processing significantly improved the polyphenolic components of sorghum, thereby enhancing their lipase inhibitory ability [154,155]. Heat treatment, microwave processing, and fermentation can improve the levels of polyphenols in foods by triggering the release of bound phenolic content into the food matrix. Nevertheless, in some cases, heat treatment and excessive microwave processing may degrade phenolic acids, thus, reducing their amounts in the final products [154,155]. Similarly, fermentation can also result in the loss of polyphenol content in foods; microorganisms used as starter cultures may use polyphenols in foods as a source of nutrients for their growth or even bioconvert them into other compounds [154]. As a result, studies have often reported conflicting results concerning the effects of processing techniques on polyphenols. Aloo et al. reported that traditional seed processing methods, such as germination, can significantly improve the polyphenolic content of foods such as buckwheat and red cabbage sprouts. The sprouts were reported to possess better inhibitory activity against lipase and alpha-glucosidase enzymes as compared to non-sprouted seeds [156]. On the other hand, emerging technologies such as ultrasound processing, high hydrostatic pressure, pulsed electric field, ultrasound, ohmic heating, high-pressure carbon dioxide processing, and irradiation are being investigated as alternatives to traditional methods for obtaining promising products with improved polyphenol contents. Compared to conventional ones, these novel technologies tend to retain most of the polyphenols and organoleptic characteristics of the final products elsewhere [154]. Thus, processing technologies have great potential to produce food products with improved polyphenol contents and anti-obesity activities. A rather in-depth analysis of the effects of these technologies on the polyphenol content of food has been discussed elsewhere [154]. In the future, the development of functional foods for the prevention and/or treatment of obesity will take advantage of processing techniques to improve the health potential of foods.

## 10. Drawbacks of Polyphenols as Phytonutrients in Foods: Benefits versus Risks

Although studies have confirmed the health benefits of polyphenols, findings from in vitro cell models, in vivo animal models, and human clinical assessments remain inconclusive. Even though experimental studies have provided extremely valuable information on the beneficial effects of dietary polyphenols, great care is required when interpreting the information obtained from these assessments. First of all, a critical problem that often compromises these studies is the safety of polyphenols. While plant bioactive compounds are generally considered safe because of their long consumption history, it is becoming increasingly apparent that some could have adverse health effects in vulnerable groups or when consumed at higher concentrations [157]. Studies on the safety of polyphenols have discovered that polyphenol consumption may be safe or unsafe depending on factors, mainly intake level and duration. Evidence indicates that taking some polyphenols for an extended period at high doses may lead to severe toxic effects. Yamakoshi et al. described a lack of adverse effects when 2–4 g/kg proanthocyanin–rich grape seed extracts were administered to rats [27]. However, in the study, Yamakoshi et al. discovered lethal doses of proanthocyanin–rich grape seed extracts at above 4 g/kg consumed for 14 days [27]. Grape seed polyphenolic extract (GSPE) was also safe in a 90-day subchronic toxicity study involving an animal model, with treatment doses ranging from 200 mg/kg/day to 215 mg/kg/day [158]. Above the amount, however, GSPE triggered adverse effects upon its consumption [158]. The recommended intake of isoflavones is 50 mg per day [159]. It was reported that the total plasma isoflavone levels range between 0.055 μmol/L and 5 μmol/L, and the daily intake from most Western diets is estimated at 0.2–5 mg/day [159]. These levels of intake are regarded as safe. However, a high intake of isoflavones, above the levels (0.2–5 mg per day), has been associated with reduced fertility and increased anti-luteinizing hormone effects [159]. Similarly, while the anti-obesity activity of catechin and its derivatives is well known, there is an increasing body of evidence that links high levels of these compounds with pro-oxidative effects due to their reaction with peroxide in the body [157]. 

Therefore, it is eminent that despite their reported positive health impacts, polyphenols may also pose a significant health risk. Meanwhile, considering the safety of phenolic compounds, as with most substances, the dose and duration of intake distinguish a poison from a remedy. Also, it is essential to recognize that the usage of these compounds depends on their benefits versus risks [157]. Thus, even though the risk of consuming high doses of polyphenols from foods is generally low, the underlying long-term effects of consuming small amounts in the body must be considered. It is crucial to note that without a complete understanding of the benefits versus risks of polyphenol intake, their addition to foods cannot be adequately justified. Thus, food producers should perform proper assessments before undertaking supplementation trials of polyphenolic compounds to uncover their safe levels. Currently, only a limited number of reports from human clinical trials are available describing the safety of polyphenols with anti-obesity effects (Table 7).

To demonstrate the bioactivity of naturally occurring compounds in foods, it is essential to define a logical chain of events that may affect their bioavailability, chemistry, and physiology. Thus, another essential aspect that should be considered when evaluating the bioactivities of polyphenols and polyphenol-rich foods is the effect of processing on the structure and bioavailability of initially present polyphenols in foods. Most polyphenols are heat-labile compounds and susceptible to light, pH variation, and enzymatic degradation. For example, the exposure of anthocyanins to high temperatures during food processing may degrade these compounds into other products (phenolic acids and phloroglucinaldehyde) [176]. The oven-baking processing can cause a degradation of chlorogenic acid [177], while boiling can significantly degrade quercetin in onions and tomatoes [178]. Hence, retaining the original phenolic compounds in fortified foods is complex and may require novel processing techniques, such as high hydrostatic pressure and irradiation, which are quite expensive [4]. 

Furthermore, polyphenol incorporation in processed foods may lead to a potential negative influence on the sensory properties (color, texture, and flavor) of food products if processing conditions are not controlled. Since most of these compounds are sensitive to heat and other environmental factors, they may react with other food components or be oxidized to form different products, such as quinones which may negatively affect food acceptability among consumers [179]. For instance, Aloo et al., and Shen et al., reported that an unusual dark color affecting the palatability of hemp-based foods is attributed to polymerization and oxidation reactions of indigenous hemp’s polyphenolic compounds and chlorophyll during processing [180,181]. So, prolonged exposure to these conditions can significantly impact polyphenols or polyphenol-rich foods, even though this effect is often ignored. This transformation process may also affect the biological functions of polyphenols, rendering them ineffective, or may even change their chemistry leading to harmful effects. Finally, the two-way interaction between gut microbiota and polyphenols leads to the biotransformation of these compounds. This process may either positively or negatively affect their bioavailability since it affects the relative abundance of beneficial gut microbiota [22].

## 11. Research Limitations 

Findings have demonstrated that polyphenols can also play a pivotal role in health. However, for several reasons, it has been challenging to establish the benefits of polyphenol consumption in humans fully. First, the polyphenol content of foods is so diverse and varies. In some cases, there is inadequate information regarding their levels of particular foods; thus, it is difficult to understand their total intake fully. Despite many mechanistic studies using pure compounds or natural plant extracts to elucidate the amount of polyphenols in food products, research has only offered limited information related to the polyphenol content, bioavailability, and extent of absorption in foods [182]. Some of these studies have also reported varying levels of polyphenols in some foods, making it challenging to add regulatory recommendations to functional food labels [183]. Conclusions on the exact level of polyphenols in regular diets remain a subject for studies. Therefore, unlike synthetic drugs, currently, there is no legislation or laws regulating polyphenol supplementation and consumption.

Second, in vitro and in vivo studies have demonstrated that dietary supplementation with plant products rich in polyphenols is a potentially viable nutritional method for the prevention of obesity. Nevertheless, the full potential of some of these foods in humans remains partially known since most of the outcomes from studies display variability and inconsistencies in clinical trials of different subjects [184]. For example, it was reported that almost all studies conducted with Asian subjects had revealed a positive outcome on the anti-obesity effects of tea extract [184]. However, clinical trials using Caucasian subjects provided mixed outcomes on the effectiveness of tea extract on obesity [184]. Therefore, human trials have often shown inconsistent results, making it difficult to make accurate conclusions on the efficacy of polyphenols against diseases. Factors such as different study designs, length of study, doses used, and choice of subjects (based on age, gender, and ethnicity) may cause this variation [2]. 

Third, in clinical trials, some polyphenols often fail to produce the results observed in either in vitro/ex vivo or in vivo studies. While the cause of this is not fully known, it is speculated that either change in mechanisms of action, the mode through which the compound is taken, or factors related to absorption may affect the efficacy of polyphenols in clinical trials [2]. Moreover, reports on the safety of the same polyphenol may also vary between in vitro, in vivo, and human clinical trials. Poulsen and colleagues indicated that while data obtained from rodent models discourage consuming high doses of resveratrol, results from human clinical trials support the safety of resveratrol consumption even at higher doses [172]. The lack of agreement between the findings from the cell, animal, and human trials raises doubts about the justification of polyphenols as a human nutritional supplement or an ingredient in food-targeting disorders [172]. 

Fourth, studies have often ignored the significance of the relationship between polyphenol doses and the duration of intake in human trials. Clinical trials need to evaluate the links between different doses of polyphenols and the duration of intake. This relationship might offer vital evidence to understand the effect of polyphenols on body weight within a specific period to avoid downstream effects resulting from an overdose due to long consumption. Another important aspect of dose that often compromises in vitro experimental models is the concentrations applied. The doses used in in vitro studies should be designed to reflect real life. However, while the tested concentrations in the in vitro studies commonly range from low μmol/L to mmol/L, the concentrations of plasma metabolites, after a normal dietary intake, hardly exceed nmol/L [185]. Thus, high doses used in vitro can “force” a conclusion on the outcome, which may not reflect in the in vivo or clinical trial results. Finally, it has been revealed that fortified foods might be more energy-dense rather than nutrient-dense [183]. This may offset any potential anti-obesogenic effects of phenolic compounds and potentially result in weight gain [183]. Thus, despite numerous studies highlighting their potential benefits, research has also shown ambiguous links between polyphenol fortification and obesity, making it difficult to recommend them effectively for food fortification [183]. 

## 12. Perspectives 

Polyphenols are essential ingredients in the food industry. Given the global rise in demand for functional foods, diversifying into these ingredients can add some resilience to the increasing impact of obesity as a nutritional approach. The following highlighted gaps need to be addressed to enhance understanding of the health benefits of polyphenols.

- In the study of plant phyto-ingredients, human and animal experiments are the most relevant in clinical nutrition but possess certain limitations. In the research on the health functions of plant metabolites, the two models have sometimes shown differences in the outcome. In most cases, the differences in the genetic composition between humans and mice are the causes of this variation [160]. Given these potential inconsistencies and to enable the public to make informed choices on polyphenol consumption, future research is needed to be more robust in clinical trials using human subjects to assess the anti-obesity effectiveness of polyphenols instead of relying on in vitro or animal findings for conclusions;
- The biological activity of polyphenols can be affected by various factors. The length of intake and mode of intake of polyphenols are key determinants of polyphenols’ biological activity [182]. However, most current studies have largely ignored the significance of the correlation between polyphenol intake and the length of intake. Furthermore, as already been mentioned, polyphenols doses administered during clinical trials are often higher than in the common diet [182]. However, only a few human studies have considered this factor in drawing a scientific substantiation for the relative claimed effects of polyphenols. In future research, it is essential to assess the relevance of these factors in the anti-obesity potential of polyphenol-rich foods to offer a verifiable claim on the health effects of polyphenols;
- Although trials have shown a correlation between polyphenol consumption and a reduction in risk factors for chronic diseases, discrepancies in explaining their positive effects have been found due to their low content in most daily diets as well as bioavailability. More studies are needed to find safe and effective methods to incorporate polyphenols in foods to improve their levels consumed in diets and to enhance the bioavailability to realize similar outcomes observed in in vitro, in vivo, and clinical trials studies. Nanocarriers are a potential technology for polyphenol encapsulation that could enhance their bioavailability, solubility and stability at their target sites in the body. In the current settings, most nanotechnology reports have addressed diseases such as cancer, but none of the studies have been reported for polyphenols targeting obesity. Future research needs to evaluate the effectiveness of encapsulated polyphenols against obesity development, both in animal and human trials, not just in vitro;
- The translation of food composition into intakes of a specific dietary compound is often achieved using food composition databases. However, for polyphenols, the approach needs to be revised since the currently available databases contain limited information regarding the diversity and concentration of phenolic compounds in plant foods. These limitations arise mainly because, unlike most nutrients, there has been a narrow systematic approach to comprehensively characterize and quantify the diverse polyphenols in plant foods using standardized analytical methods. Moreover, much of the available information has been assembled solely from heterogeneous sources, in which the original food sampling and description are still being investigated. Furthermore, the complexity arises due to the uneven distribution of polyphenolic compounds in different parts of plants and the loss of specific polyphenols during food processing. For example, in apples, quercetin is mainly found in the peel; however, peeled fruit contains no quercetin. Similarly, most polyphenols in wheat grain are located primarily in the outer layers and are usually lost during the refining process of the flour. Further research is required to expand our current knowledge regarding alternative dietary assessment methods that could help overcome these challenges;
- Finally, to establish firm evidence for the health effects of dietary polyphenol consumption, it is essential to have quantitative information regarding their dietary intake on food labels. This is particularly essential for enriched foods or foods that are known to contain high amounts of these compounds. The usefulness of such information is that consumers can have a planned dietary intake based on specific foods. Thus, similar to other known nutrients and drugs, in the future, food records and labels for polyphenols need to be developed to allow consumers to quickly assess total intake and required precautionary measures accurately.

## 13. Concluding Remarks

Evidence from cellular, animal and human studies indicates that consuming polyphenols in diet or as a supplement enhances protection against obesity. Anti-obesity activities of polyphenols may occur through mechanisms including enzyme inhibition, suppression of neuro-hormones related to food intake and satiety, and induction of mitochondrial biogenesis. Since polyphenols demonstrate great anti-obesity potential, screening for polyphenol-rich foods with anti-obesity effects but fewer adverse reactions in humans will likely offer a novel and effective therapeutic strategy to fight obesity. A comprehensive safety evaluation is needed to ascertain consumer safety of polyphenols consumption in foods. Rigorous assessment of these compounds in human trials will provide evidence-based results that will facilitate the incorporation of natural phenolic compounds in functional foods or as supplements to help fight the growing problem of obesity.

## Figures and Tables

**Table 1:** Safety of anti-obesity drugs in clinical studies.

Drug	Country	Participants	Dose	Reported Adverse Effect	References
**Common Drugs**					
*For short-term use*					
Diethylpropion	Brazil	*N* = 69	50 mg per day for 6 months	Dry mouth and insomnia	[8]
Mazindol	USA	*N* = 85	1–3 mg per day for 6 weeks.	Dry mouth, nausea, decreased appetite, fatigue, heart rate, and constipation	[9]
Phendimetrazine	USA	*N* = 13	140–210 mg per day for 7-days	No observable adverse effects	[10]
Phentermine	Korea	*N* = 74	30 mg per day for 12 weeks	Dry mouth and insomnia	[11]
*For long-term use*					
Orlistat	Japan	*N* = 100	60 mg three times daily for 24 weeks	Oily spotting and flatus with discharge	[12]
Topiramate ER	Canada, Europe and South Africa	*N* = 646	192 mg per day for 24 weeks.	Paresthesia and central nervous system-related effects.	[13]
Lorcaserin	USA	*N* = 604	10 mg per day for 12 months	Headache, nasopharyngitis, nausea, and back pain	[14]
Sibutramine	USA	*N* = 61	20 mg once daily for 12 weeks	No adverse effect	[15]
Naltrexone/Bupropion ER	USA	*N* = 1650	Naltrexone 32 mg per day plus bupropion 360 mg per day 56 weeks	Headache, constipation, nausea, dizziness, vomiting, and dry mouth	[16]
**GLP-1 receptor agonists**					
*For short-term use*					
Exenatide	China	*N* = 681	2 mg per day for 10 weeks	Nausea, dyslipidemia, and vomiting	[17]
Lixisenatide	France	*N* = 484	20 µg per day for 24 weeks	Acute pancreatitis	[18]
*For long-term use*					
Liraglutide,	Australia, Belgium, the UK, the USA, Russia, Israel, and Germany	-	30 mg per day for 52 weeks	Gastrointestinal symptoms, primarily nausea	[19]
Albiglutide	USA	*N* = 155	30 mg per week for 52 weeks	Diarrhea, nausea, and vomiting	[20]
Dulaglutide	Not reported	*N* = 807	1.5 mg or 0.75 mg per day for 52 weeks	Nausea, diarrhea, and vomiting	[21]

**Table 2:** Polyphenol-enriched food products targeting obesity.

Polyphenol-Rich Extract	Enriched Product	Experimental Model	Main Observation	References
A mixture of polyphenol-rich extracts from green tea, grape seed, and baobab fruit	White bread	Clinical trial	Improved insulin sensitivity	[36]
Catechins, chlorogenic acids, and hydroxytyrosol	Cooked ham	In vivo	Decreased fat accumulation (23.08% reduction)	[37]
Extracts of pomegranate peels and pomegranate peels (rich in ellagitannins, gallic acid, and ellagic acid)	Cookies	In-vitro	Inhibited α-glucosidase, α-amylase, and lipase activities	[38]
Green tea extract (containing catechins)	Bread	Clinical trial	Improved body weight, waist circumference	[39]
Green tea polyphenols	Bread	In vivo	Significantly suppressed body weight gain	[40]
Proanthocyanidins from carob fruit extract	Meat	In vivo	Decreased accumulation of liver fats	[41]
Polymerized-polyphenol extract from oolong tea	Oolong tea	Clinical trial	Improved body lipid accumulation	[42]

The table describes the polyphenol-enriched food products developed to target obesity in either experimental animal or human clinical trials. The effect of the foods on obesity parameters was evaluated following the intake of polyphenol-enriched.

**Table 3:** Cellular and animal studies on the potential effects of polyphenols and polyphenol-rich extracts on obesity.

Polyphenol	Model	Treatment Dose	Key Observation	References
Polyphenols-rich blueberry extract	Mice	Mice were provided with 15.6 mg/kg BW per day for 12 days	Inhibited body weight gain and reverted lipid metabolism to normal.	[76]
Rambutan seeds extract (containing alkaloid, terpenoid, triterpenoid, and flavonoids)	3T3-L1 cell line	Cells were treated with varying concentrations of the extracts (10 and 50 µg/mL)	Decreased triglyceride levels. Inhibited glucose-6-phosphate dehydrogenase (G6PDH) which promote adipogenesis	[73]
Procyanidin-rich grape seed extract (GSPE)	3T3-L1	Cells were treated with 140 mg/L GSPE (dissolved in water) for 24 hr on days 0, 2, or 4.	Reduced by 32% triglyceride content in cells treated at day zero. Downregulated genes responsible for preadipocyte differentiation but elevated preadipocyte factor-1 (Pref-1).	[75]
Polyphenol-rich cranberry extracts	Mice	Mice were provided with 0.75% (*w*/*w*) of polyphenol-cranberry-rich extract per day for 16 weeks.	Elevated energy expenditure and brown adipose tissue thermogenesis.	[77]
Polyphenol-rich totum-63 extract	Mice	Mice were fed with a high-fat diet for 12 weeks, followed by supplementation with Totum-63 (2.7% *w*/*w*) for 4 weeks.	Decreased body weight and fat mass. Increased expression of insulin receptor β and insulin-induced phosphorylation of PKB in skeletal muscle, white adipose tissue (WAT), and brown adipose tissue (BAT), thereby inducing thermogenesis.	[78]
Polyphenol-rich extracts from *Antirhea borbonica*, *Doratoxylon apetalum* and *Gouania mauritiana*	3T3-L1	Cells were treated with polyphenol-rich from the respective extract (0–200 μM GAE) for 24, 48, and 72 h	Inhibited obesity-induced inflammation and oxidative stress	[79]
Anthocyanin-rich blueberry extract	Mice.	Mice were fed with 50–200 mg/kg per day for 8 weeks	High doses of 200 mg/kg reduced body weight by 19.4%, while 50 and 100 mg/kg doses did not significantly affect body weight.	[80]
Cyanidin 3-*O*-β-d-glucoside (C3G)-rich blackberries	Rats	Rats were given 10% (*w*/*w*) of C3G-rich blackberry	Reduced overall body weight	[81]
Acacia-rich polyphenols (containing catechin-like flavan-3-ols, such as robinetinidol and fisetinidol)	Mice	Mice were provided with a high-fat diet supplemented with 2.5–5.0% (*w*/*w*) of acacia polyphenol extract for 7 weeks.	Decreased body weight and also elevated the mRNA expression of energy expenditure-related genes	[82]
Anthocyanins-rich black soybean extract	3T3-L1 cells	Cells were treated with anthocyanins-rich black soybean extract (at a concentration of 12.5–50 μg/mL)	Inhibited the proliferation of both preconfluent preadipocytes and mature postconfluent adipocytes	[74]

BW, body weight; IL-6, Interleukin 6; TNF, tumor necrosis factor-alpha; AMPK: adenosine monophosphate-activated protein kinase; (iWAT/eWAT, inguinal/epididymal white adipose tissue; PKB, protein kinase B.

**Table 4:** Anti-obesity effects of polyphenol-rich extracts in clinical trials.

Polyphenol	Subject	Country	Study Objective	Intervention	Key Observations	References
Polyphenol-rich green tea extract	*N* = 100, Women, age: 16–60 years BMI: >27 kg/m^2^	Taiwan	To examine the effect of green tea extract on obesity.	The subjects consumed green tea containing (491 mg of catechins containing 302 mg EGCG per day) for 12 weeks.	0.3% reduction in body weight (Equiv. 0.15 kg) after 12 weeks of treatment. Significantly reduced triglyceride levels.	[84]
Polyphenol-rich chocolate (contained mainly epicatechin)	*N* = 1017, Men and women aged: 20 to 85 years	USA	To evaluate the effect of chocolate rich in phenolic compounds on body mass index.	Subjects ate chocolate, with a mean intake of 2.0 (2.5) times/week and exercised 3.6 (3.0) times/week.	Improved BMI.	[85]
Yerba Mate (*Ilex Paraguariensis*) (rich in (quercetin rutin, chlorogenic and caffeic acids)	*N* = 15 BMI < 35 and ≥ 25 kg/m^2^ and waist-hip ratio (WHR) ≥ 0.90 for men or ≥ 0.85 for women	Korea	To investigate the efficacy of Yerba Mate supplementation against obesity.	The subjects were given 13 g/day of Yerba Mate capsules for 12 weeks.	Decreased BMI (*p* = 0.036), body mass fat (*p* = 0.030), and waist-hip ratio (*p* = 0.004).	[86]
Soya isoflavones	*N* = 100, postmenopausal women age: 50–70 years BMI 28–40 kg/m^2^	Canada	To assess the combined effect of exercise and soy isoflavones on obesity.	Subjects consumed a 70 mg/day dose of isoflavones for 12 months.	Decreased trunk fat mass and increased lean body mass.	[87]
Mixture of polyphenols	*N* = 573, 277 men, 296 women, age: 66.2–68.3 years BMI > 30 kg/m^2^	Spain	To assess the associations between total polyphenol and obesity parameters among the elderly after a long period of polyphenol intake (measured by overall urinary polyphenol level).	Participants known to consume foods rich in polyphenols were recruited and followed up for 5 years. Spot urine samples were collected and analyzed for total polyphenols, and obesity indicators were measured.	Increased consumption of dietary polyphenols was associated with improved BMI after 5 years of consumption.	[88]
Citrus polyphenolic extract of red-orange, grapefruit, and orange (Sinetrol-XPur)	*N* = 95, 55 women and 40 men, age: 22–45 years BMI 26–29.9 kg/m^2^	France	To investigate appropriate polyphenolic-rich combinations that would help reduce body fat, inflammation, and oxidative stress in overweight subjects.	Subjects consumed two capsules of citrus polyphenolic extract containing orange, grapefruit, sweet orange, and guarana daily for 12 weeks.	Reduced abdominal fat and overall body weight.	[83]
Polyphenol-rich green tea extract	*N* = 35, Men and women, mean, age 42.5 ± 1.7 years BMI: 36.1 ± 1.3 kg/m^2^	USA	To compare the effects of green tea polyphenols with controls on body weight and safety parameters in obese subjects.	Subjects took either four cups of decaffeinated green tea beverage or two capsules of green tea extract containing either 28 mg or 870 mg of catechins (GC, GCG, EC, ECG, EGC, and EGCG) daily for 8 weeks.	Decreased body weight. Improved LDL-cholesterol level	[89]
Licorice flavonoid oil	*N* = 22, men and women, age: 20–53 years BMI: 25.0–36.0 kg/m^2^	USA	To investigate the effect of licorice flavonoid oil supplementation on obesity-related health markers.	Subjects consumed three capsules of licorice flavonoid oil, 300 mg per day for 8 weeks.	Inhibited the total cholesterol level. Decreased total triglycerides.	[90]

LDL, low-density lipoprotein; HDL, high-density lipoprotein, LFO, licorice flavonoid oil; BMI, body mass index; GC, gallocatechin; GCG, gallocatechin-3-gallate; EC, epicatechin; ECG, epicatechin-3-gallate; EGC, epigallocatechin; EGCG, epigallocatechin-3-gallate.

**Table 5:** Studies elucidating the anti-obesity effects of short-chain fatty acids (SCFA) in animal and human models.

Model	Experimental Procedure	Observation	References
Mice	Mice were fed with a high-fat diet supplemented with sodium acetate, sodium propionate, sodium butyrate or their mixture (ratio at 3:1:1)	Caused changes in the bacterial community: reduced the proportion of *Firmicutes* and increased *Bacteroidetes.*	[122]
Human	Participants received a daily dietary supplement of 24 g inulin (source of SCFA) for two investigation days, with at least 5 days of washout	Improved β-cell function with increased insulin secretion. No effects on plasma triglycerides, or free glycerol.	[123]
Mice	The mice were fed diets containing sodium acetate, sodium propionate or sodium butyrate at 5% (*w*/*w*).	Induced reduction in body weight and stimulated insulin sensitivity.	[124]
Mice	Animals were fed a high-fat diet supplemented with 5% acetate or propionate (in the presence of 5% cellulose).	SCFA lowered hepatic triglycerides and improved insulin sensitivity.	[125]
Human	Participants (*n* = 441) were recruited and examined for their fecal SCFA, and related markers of obesity were analyzed.	Higher SCFA in fecal excretion was associated with gut modulation effects.	[126]

**Table 6:** Metabolites from the digestion of polyphenols by intestinal microbiota and their reported anti-obesity effects.

Polyphenol	Gut Bacteria Involved	Model	Main Metabolite	Major Observation	References
Epicatechin	*Eubacterium sp., Bifidobacterium sp., Lactobacillus, anaerobic cocci, and, Fusobacterium spp.*	In vitro study (human feces)	5-(3,4-dihydroxyphenyl)-γ-valeric acid,3-(3-hydroxyphenyl)propionic acid,4-hydroxyphenyl acetic acid	-	[129]
Quercetin	*Eubacterium ramulus, Eggerthella sp.*	Animal study (urine)	4-ethylphenol, Benzoic acid,4-ethylbenzoic acid	Inhibitory effects on α-amylase enzyme	[130]
Epicatechin	-	Animal study (urine)	1,3,5-Trimethoxybenzene	Inhibit adipocyte differentiation	[130,131]
Quercetin	*Bacteroides*	In vitro study (humans feces)	Hydroxyphenylacetic acid derivatives	Gut microbiota modulation	[132]
Rutin	*Bacteroides*	In vitro study (humans feces)	3,4-dihydroxyphenylacetic acid	Gut microbiota modulation	[132]
Isoflavone	*Streptococcus intermedius, Bifidobacterium spp., Bacteroides ovatus, Streptococcus intermedius, Escherichia coli*	In vitro study (human urine)	Dihydrodaidzein (DHD), tetrahydrodaidzein (THD), equol, and *O*-DMA	-	[25]
Flavan-3-ols	*Clostridium coccoides, Bifidobacterium spp.*	In vitro study (human feces)	Dihydroxyphenylpropionic (dihydrocaffeic) acid and 3,4-dihydroxybenzoic (protocatechuic) acid (PCA)	Inhibit pancreatic lipase activities	[25,133]
Genistin,	*No specific bacteria reported*	In vitro study (human and animal feces)	4-hydroxyphenyl-2-propionic acid and 1,3,5-trihydroxybenzene	-	[134]
Anthocyanin	*Lactobacillus spp.*	In vitro study (human feces)	Gallic acid, syringic acid and p-coumaric acid	Inhibition of preadipocytes growth	[135,136]
Ellagic acid	*Gordonibacter urolithinfaciens*	In vitro study (human feces)	Urolithins	Inducing thermogenesis in brown adipose tissue (BAT) and inducing browning of white adipose tissue (WAT).	[137,138]
Ellagitannins	*Gordonibacter urolithinfaciens*	In vitro study (human feces)	Urolithins	Inducing thermogenesis in brown adipose tissue (BAT) and inducing browning of white adipose tissue (WAT	[139]
Naringenin	*No specific bacteria reported*	In vitro study (rat feces)	Phenylacetic acid, protocatechuic acid	-	[140]
Chlorogenic acid	*No specific bacteria reported*	In vitro study (human feces)	3-(3-hydroxyphenyl)-propionic acid	-	[141]
Resveratrol	*Slackia equolifaciens, Adlercreutzia equolifaciens*	In vivo and in vitro (human feces)	Dihydroresveratrol, and lunularin	-	[25,142]
Baicalin	*No specific bacteria reported*	In vitro study (human feces)	Baicalein and oroxylin A	Enhances pAKT, PGC-1α and UCP1	[143,144]
Apigenin	*No specific bacteria reported*	Animal study (urine)	P-hydroxyphenyl acetic acid, P-hydroxycinnamic acid, P-hydroxybenzoic acid	Inhibition of adipogenesis	[145,146]

**Table 7:** Studies investigating the safety of polyphenols in human clinical trials.

Polyphenol	Subject Description	Dosage	Side Effect	References
Resveratrol (RV)	40 healthy volunteers aged 18–80 years	Participants took oral resveratrol (single doses of 0.5, 1, 2.5, or 5 g) per day for 1 week.	An intake of up to one dose of 5 g of resveratrol was safe, with only minor adverse events in some cases.	[160]
Sinetrol-XPur (polyphenolic citrus dry extract)	95 healthy overweight volunteers (55 women and 40 men), age: 22–45 years, BMI: 26–29.9 kg/m^2^	Subjects consumed two capsules of citrus polyphenolic extract containing orange, grapefruit, sweet orange, and guarana for 12 weeks	Mild effect, such as a slight increase in cardiac rate, was observed	[83]
Resveratrol	24 overweight patients, median age: 66.5 years	Participants took 5.0 g resveratrol per day for 4 months	Serious adverse effects, including nausea, diarrhea, vomiting, and fatigue	[161]
Resveratrol	62 participants (men and women)	Participants consumed 250 mg daily for 3 months	Improved glycemic control with no observable side effects	[162]
Epicatechin	age:18–50 years BMI: 19–30 kg/m^2^	Subjects consumed 250 mg of cocoa flavanols; 40 mg of epicatechin for 14 days	Improved body weight parameters with no adverse events observed	[163]
Ellagitannin	64 overweight individuals, age: 40–70 years, BMI: 25–32 kg/m^2^	Subjects consumed 710 mg per day of a pomegranate ellagitannin-enriched polyphenol extract.	No serious adverse events on the subject upon the intake	[164]
Polyphenon E	40 healthy participants with Fitzpatrick skin types II or III, age: ≥18 year	Participants took 800 mg polyphenol E once per day for 2 weeks	Adverse events, including stomach upset, nausea, heartburn, stomach pain, dizziness, headache, and muscle pain, were observed during the 4-week treatment period	[165]
Polyphenols-rich green tea	17 healthy volunteers, age: 41 ± 9 years BMI 26.7 ± 3.3 kg/m^2^,	Participants consumed tea containing 119 mg polyphenols (epicatechin, 5 mg; epigallocatechin, 47 mg; epigallocatechingallate, 25 mg; epicatechingallate, 14 mg; gallocatechingallate, 8 mg; catechingallate, 3 mg; catechin, 1 mg; gallocatechin, 9 mg; and ellagic acid, 7 mg) and 19 mg caffeine per day for 3 weeks	The daily consumption of green tea polyphenols, even at high dose levels, was safe. No effects on cardiovascular risk biomarkers screened	[166]
Curcumin	10 healthy male volunteers aged 20–26 years. Weight: 50–75 kg	Participants consumed beverages formulated with 2 g of curcumin	Improved serum concentration without any toxic effect observed	[167]
EGCG and α-glucosyl hesperidin (gH)	60 healthy males and females aged: 30–75 years	Subjects were given green tea, 178 mg gH and 146 mg EGCG per day for 12 weeks	The amount of EGCG and gH consumed effectively reduced body weight with no adverse effects	[168]
Juçara fruit polyphenols	35 adults known to be obese (men and women) aged 31–59 years. BMI: 30–39.9 kg/m^2^	Subjects consumed 5 g of pulp powder per day for 6 weeks	No toxic effects were observed. Authors’ conclusion: safe for consumption	[169]
Hibiscus and lemon verbena polyphenols	54 overweight subjects, age: 30–75 years BMI: 25–34.9 kg m^−2^	500 mg of *Lippia citriodora* (35%) and *Hibiscus sabdariffa* (65%) per day for 2 months was taken by the studied subjects	No toxic effects were reported	[170]
Green tea (containing majorly, catechins)	35 subjects with obesity and metabolic syndrome	Subjects consumed green tea (four cups/day) or green tea extract (two capsules/day) for 8 weeks.	Improved obesity-related parameters without any observable side effect	[89]
*Lippia Citriodora and Hibiscus Sabdariffa* extract *(Lc-Hs)*	33 Volunteers (male and female), age: 18–65 years, BMI: 25–34.9 kg/m^2^	Oral administration of two capsules/day, each capsule containing 250 mg for 60 days	The supplementation with the Lc-Hs extract decreased appetite sensation; no toxicity was observed upon consumption	[171]
High dose of resveratrol	24 obese but healthy volunteers (men), age: 18–70 years, BMI >30 kg/m^2^	Subjects consumed 500 mg/day of resveratrol for 4 weeks	Improved key obesity indicators (total and lean body mass, total body fat mass, or visceral and abdominal subcutaneous fat). No adverse effects were observed.	[172]
Catechin-enriched green tea	33 obese subjects (18 men, 15 women), age: 20–65 years, waist circumference: ≥80 cm (women) or 90 cm (men)	Subjects drank a 350-mL bottle of beverage after lunchtime within 30 min daily for 12 weeks.	Adverse events such as changes in stools, abdominal discomfort, and appetite were associated with catechin-enriched green tea consumption	[173]
Acacia bark-derived proanthocyanidins		Participants took proanthocyanidins derived from acacia bark (245 mg/day for 12 weeks)	No side effects or adverse events were observed upon the consumption	[174]
Gallic acid	105 healthy subjects age: 18–60 years, BMI: 25–35 kg/m^2^	Subjects took a 300 mg/1.2 g NT-GA combination or 600 mg/2.4 g/day NT-GA for 24 weeks.	NT-GA consumption was well tolerated but was ineffective in causing weight loss or limiting food intake	[175]

RV, Resveratrol; GA, gallic acid; EGCG, epigallocatechin gallate; BMI, body mass index; NT is an herbal supplement derived from a water extract of rhubarb, ginger, astragalus, red sage, and turmeric.

## Supplementary Materials

The following supporting information can be downloaded at: https://www.mdpi.com/article/10.3390/antiox12020416/s1.
