# Supplementary material for: Insights on Dietary Polyphenols as Agents against Metabolic Disorders: Obesity as a Target Disease
Source: Antioxidants (Basel). 2023 Feb 8;12(2):416. doi: 10.3390/antiox12020416 (PMC9952395; doi:10.3390/antiox12020416)
Supplement: Supplementary file 1 [file antioxidants-12-00416-s001.zip › antioxidants-2214269-supplementary.pdf]

Online material

**Insights on Dietary Polyphenols as Agents against Metabolic Disorders: Obesity as a Target Disease**

**Simon-Okomo Aloo[1], Fred Kwame Ofosu[1], Nam-Hyeon Kim[1], Sheila M Kilonzi[2,] and Deog-Hwan Oh[1,*]**

[1]Department of Food Science and Biotechnology, College of Agriculture and Life Sciences, Kangwon National University, Chuncheon, Gangwon-do 24341, Republic of Korea

[2] Department of Food Science and Nutrition, School of Agriculture and Biotechnology, Karatina University, Karatina 10101, Kenya

"Online Supplementary Material"

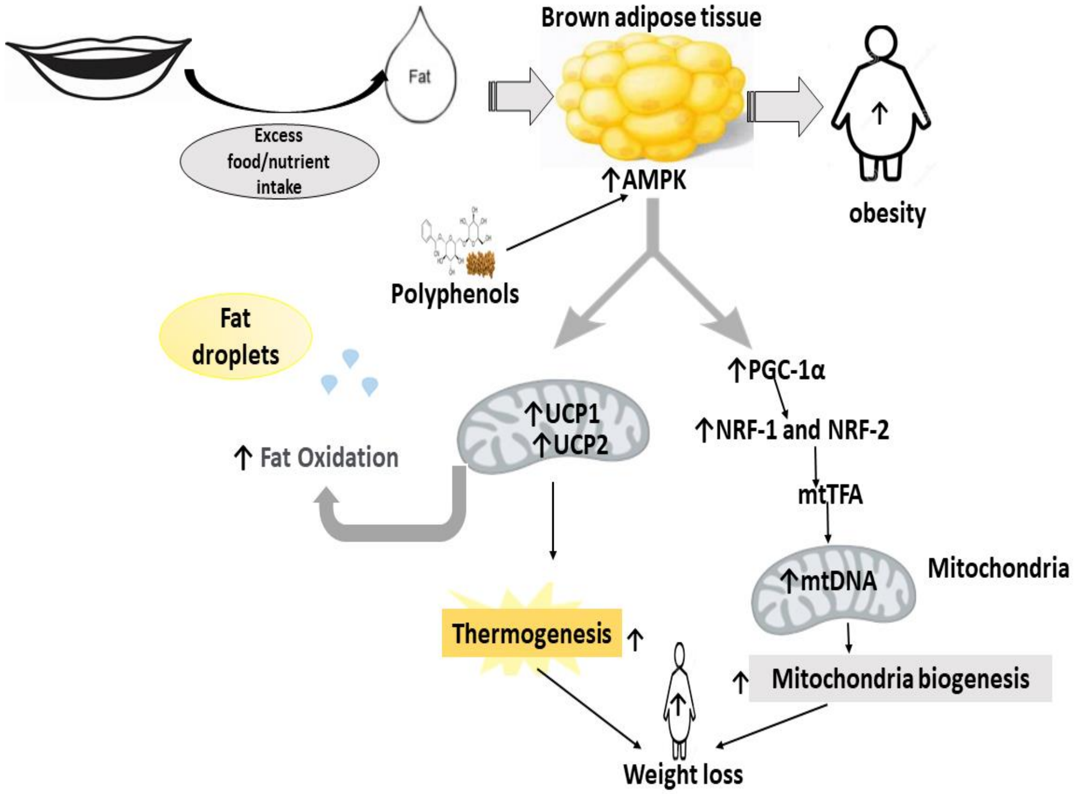


Supplementary Figure S1. The role of dietary polyphenols on inducing thermogenesis and mitochondrial biogenesis through the AMPK pathway. PGC-1$\alpha$ is a transcriptional regulator that induces mitochondrial biogenesis by activating various other transcription factors, such as NRF-1 and NRF-2, which activate mtTFA[1]. The mtTFA drives transcription and replication of mtDNA. PGC-1$\alpha$ is regulated by different key factors involved in mitochondrial biogenesis, including AMPK. The AMPK can also act as an energy sensor of the cell and works by regulating key proteins such as UCP1 and 2 involved in thermogenesis [2]. PGC-1$\alpha$ (peroxisome-proliferator-activated receptor $\gamma$ co-activator-1$\alpha$); NRF-1 and NRF-2 (nuclear respiratory factor 1 and nuclear respiratory factor 2); mtTFA (Mitochondrial transcription factor A); AMPK (AMP-activated protein kinase); mtDNA (mitochondrial DNA); ↑ (Up-regulation

Supplemental Table S1. Lipase inhibitory activities of polyphenols and polyphenol-rich extracts

| **Polyphenols/polyphenol-rich extract** | **Dose** | **Inhibition rate** | ***In-vivo/in-vitro*** | **Reference** |
|---|---|---|---|---|
| (−)-Epigallocatechin 3-O-gallate (EGCG) from Oolong tea | 0.01 to 10 µg/ml | $IC_{50}$ of 0.349 Mm | *in-vitro* | [3] |
| Black tea polyphenols | 500 or 1000 mg/kg body weight | $IC_{50}$ of 15.5 | *In-vivo* | [4] |
| Walnut polyphenols | 0 µg/mL to 350 µg/Ml | $IC_{50}$ of 163 µg/ml | *in-vitro* | [5] |
| black chokeberry fruit polyphenols | 13, 16 and 18 mg/Ml | 60.31 µg/ml | *In-vitro* | [6] |
| Kaempferol 3-O-$\alpha$-L-arabinopyranosyl-5-O-$\alpha$-L-rhamnopyranoside from *Vicia faba L* | 400 and 800 µg/mL, | 53% | *In-vitro* | [7] |
| Curcumin | 100 Ml | $IC_{50}$ value 250Mm | *In-vitro* | [8] |
| Chlorogenic acid | 20 mg/kg | 745 U/L | *In-vivo* | [9] |
| 24hr fermented isoflavones | 50 µg/ml | 63.6% | *In-vivo* | [10] |
| Quercetin | 40 Mm | 80% | *In-vivo* | [11] |
| Pistachio green hull tannins | 490 µg | $IC_{50}$ value 2.26 mg/ml | *In-vivo* | [12] |

Supplementary Table S2. Fatty acid synthase (FAS) inhibitory activities of polyphenols and polyphenol-rich extracts

| **Polyphenols/polyphenol-rich extract** | **Dose** | **Inhibition rate** | ***In-vivo/in-vitro*** | **Reference** |
|---|---|---|---|---|
| Resveratrol | 0-25 μg/ml | $IC_{50}$ value 11.1 μg/ml | *In-vitro* | [13] |
| Resveratrol | 45 mg/kg (6 weeks) | 46nmol/min/mg protein | *In-vivo* | [14] |
| Mulberry polyphenols | 0.5-2% (w/w) extracts with high fat diet (12 weeks | - | *In-vivo* | [15] |
| Olive oil polyphenols | 10, 25, 50 and 100 μM (72 h ) | - | *In-vitro* | [16] |
| Tea polyphenols (theaflavin and (−)-epigallocatechin 3-gallate) | 30 μM | 52-87% | *In-vitro* | [17] |
| Lotus root polyphenols | 0.5% lotus root extract containing 892 mg/g for 3 weeks | 6-9nmol/min/mg protein | *In-vivo* | [18] |
| Oolong tea polyphenols | 400 or 800 mg/kg (6 weeks) | - | *In-vivo* | [19] |
| Ginger polyphenol | 10-40 μM (2 h) | - | *In-vitro* | [20] |

Supplementary Table S3. The effects of polyphenols on the body thermogenesis and mitochondrial biogenesis

| Polyphenols | Mode | Dose | Effect | Reference |
|---|---|---|---|---|
| Resveratrol | Neural progenitor cells of mice | 2.5 mg/kg, BW (15 days) | ↑SIRT3 ↑Parkin | [21] |
| Resveratrol | Human coronary arterial endothelial cells | 10 μmol/l (24hr) | Nrf-1 PGC-1$\alpha$ | [22] |
| Epigallocatechin-3-gallate | Obese mice | 0.2% EGCG (w/w) (8 weeks) | ↑PGC-1$\alpha$ ↑ NRF1 ↑ Tfam | [23] |
| Quercetin | Liver tissue of mice | 100 mg/kg (15 weeks) | ↑AMPK, ↑Parkin | [24] |
| Curcumin (turmeric) | Cancer and HUVEC cells | 10 μM (1–24 h) | ↑Mitophagy ↓mTOR ↑LC3-II | [25] |
| Myricetin, gallic acid, caffeic acid, and catechin | 3T3-L1 preadipocytes | Various concentration of individual polyphenols (24-48hr) | ↑PGC-1$\alpha$ | [26] |
| Apple polyphenols | Obese mice | 5 g $kg^{-1}$ APs supplementation for week 10 | ↑UCP1 ↑mRNA ↑PRDM16 ↑PGC-1$\alpha$ | [27] |
| p-Coumaric acid | C3H10T1/2 cells | 0, 1, 10, and 100 μM (6 days) | ↑UCP1 | [28] |
| Epigallocatechin gallate | Obese mice | 500 mg/kg (4 weeks) | ↑UCP1 | [29] |
| Gentisic acid | C3H10T1/2 cells | 0 μM to 10 μM for 24 h | ↑UCP1 | [30] |

Tfam, mitochondrial transcription factors A, UCP1, uncoupling protein 1; NRF1, nuclear respiratory factor-1; SIRT3, sirtuin-3; mTOR, target of rapamycin; LC3, light chain 3; ↑, up-regulation; ↓, down regulation

Supplementary Table S4: Studies evaluating the effects of polyphenols on gut microbiota composition in obesity

| **Polyphenol** | **Model** | **Treatment dose** | **Effect on gut microbiota** | **Reference** |
|---|---|---|---|---|
| Green tea polyphenols | Mice | 0.2% by weight tea polyphenols per day for 8 weeks | Improved *Bacteroidetes* to *Firmicutes* ratios | [31] |
| Berries-rich polyphenols | Mice | 200 mg/kg, BW per day for 8 weeks | Improved abundance of *Akkermansia muciniphila, Dubosiella newyorkensis*, and *Angelakisella* | [32] |
| Blueberry anthocyanin | Mice | 2% by weight blueberry extract per day for 24 weeks | Triggered growth of *Lachnoclostridium, Roseburia*, and *Clostridium_innocuum_group* | [33] |
| Olive oil phenolic compounds | Human trial | 25 mL per day for 3 weeks | Increased numbers of *bifidobacteria* | [34] |
| Polyphenol-rich cranberry | Mice | 200 mg/kg per day for 8 weeks. | Increased the proportion of the mucin-degrading bacterium, *Akkermansia* | [35] |
| Fruits and vegetable polyphenols | Human trial | Fruits or vegetables with high-flavonoid or low flavonoid taken to increase polyphenol intake by 2, 4, and 6 portions after 6 weeks. | Modulated composition by increasing *Clostridium leptum-Ruminococcus bromii/flavefaciens* and decreasing *Clostridia* | [36] |
| *Trans*-resveratrol and quercetin | Mice | 15 mg/kg, BW per *trans*-resveratrol and 30 mg/kg, BW per quercetin for 6-weeks | Co-administration of *trans*-resveratrol and quercetin elevated *Bacteroidetes* to *Firmicutes* ratio and inhibited the growth of obesogenic bacteria (*Erysipelotrichaceae*, *Bacillus*, and *Eubacterium glindroides*) | [37] |
| Resveratrol and quercetin | Mice | Combination of quercetin (30 mg, BW per day) and resveratrol (15 mg, BW per day) for 10 weeks | Increased the *Bacteroidetes* to *Firmicutes* ratio and reduced the amounts | [38] |

Supplementary Table S5. Applications of nano and micro-encapsulation to improve the bioavailabilty and delivery of polyphenols

| Polyphenols | Nano-particle involved | References |
|---|---|---|
| (+)-catechin | Chitosan–tripolyphosphate, Chitosan-caseinophosphopeptides | [39, 40] |
| Quercetin | Guar gum | [41] |
| Green tea Polyphenols | Chitosan, Gelatin, Milk proteins | [42] |
| Tea polyphenols | Chitosan | [43] |
| Curcumin | Chitosan | [43] |
| Anthocyanins | Cyclodextrins, Shellac and shellac/hydroxypropyl methylcellulose | [44, 45] |
| Tannic acid | Islet | [46] |
| Proanthocyanidins | Apo-red bean ferritin, Chitosan | [47] |
| Cocoa flavanols and phenolic acids | High-amylose maize starch | [48] |
| Resveratrol | Emulsion-based | [49] |
| Resveratrol | Liposomes/Niosomes | [50] |
| Carob pulp polyphenolics extrac | Polycaprolactone | [51] |
| Epigallocatechin gallate | Protein-based nanoparticles | [52] |
| Hesperidin | Sodium carboxymethyl cellulose | [53] |

## References


1. Brown, G.C., et al., *Regulation of mitochondrial biogenesis.* Essays in biochemistry, 2010. **47**: p. 69-84.
2. Pollard, A.E., et al., *AMPK activation protects against diet-induced obesity through Ucp1-independent thermogenesis in subcutaneous white adipose tissue.* Nature metabolism, 2019. **1**(3): p. 340-349.
3. Nakai, M., et al., *Inhibitory effects of oolong tea polyphenols on pancreatic lipase in vitro.* Journal of Agricultural and Food Chemistry, 2005. **53**(11): p. 4593-4598.
4. Uchiyama, S., et al., *Prevention of diet-induced obesity by dietary black tea polyphenols extract in vitro and in vivo.* Nutrition, 2011. **27**(3): p. 287-292.
5. Shi, D., et al., *Walnut polyphenols inhibit pancreatic lipase activity in vitro and have hypolipidemic effect on high-fat diet-induced obese Mice.* J. Food Nutr. Res, 2014. **2**: p. 757-763.
6. Sosnowska, D., et al., *Inhibitory effect of black chokeberry fruit polyphenols on pancreatic lipase–Searching for most active inhibitors.* Journal of Functional Foods, 2018. **49**: p. 196-204.
7. Allam, A.E., et al., *Effect of polyphenols from Vicia faba L on lipase activity and melanogenesis.* Natural product research, 2018. **32**(16): p. 1920-1925.
8. Kim, T.H., et al., *Enhancement of pancreatic lipase inhibitory activity of curcumin by radiolytic transformation.* Bioorganic & medicinal chemistry letters, 2011. **21**(5): p. 1512-1514.
9. Ohkawara, T., H. Takeda, and J. Nishihira, *Protective effect of chlorogenic acid on the inflammatory damage of pancreas and lung in mice with l-arginine-induced pancreatitis.* Life sciences, 2017. **190**: p. 91-96.
10. Choi, I., et al., *Anti-obesity activities of fermented soygerm isoflavones by Bif idobacterium breve.* Biofactors, 2007. **29**(2-3): p. 105-112.
11. Gatto, M.T., et al., *Antimicrobial and anti-lipase activity of quercetin and its C2-C16 3-O-acyl-esters.* Bioorganic & medicinal chemistry, 2002. **10**(2): p. 269-272.
12. Noorolahi, Z., et al., *Tannin fraction of pistachio green hull extract with pancreatic lipase inhibitory and antioxidant activity.* Journal of food biochemistry, 2020. **44**(6): p. e13208.
13. Liang, Y., W. Tian, and X. Ma, *Inhibitory effects of grape skin extract and resveratrol on fatty acid synthase.* BMC complementary and alternative medicine, 2013. **13**(1): p. 1-8.
14. Gómez-Zorita, S., et al., *Resveratrol attenuates steatosis in obese Zucker rats by decreasing fatty acid availability and reducing oxidative stress.* British Journal of Nutrition, 2012. **107**(2): p. 202-210.
15. Peng, C.-H., et al., *Mulberry water extracts possess an anti-obesity effect and ability to inhibit hepatic lipogenesis and promote lipolysis.* Journal of agricultural and food chemistry, 2011. **59**(6): p. 2663-2671.
16. Notarnicola, M., et al., *Effects of olive oil polyphenols on fatty acid synthase gene expression and activity in human colorectal cancer cells.* Genes & nutrition, 2011. **6**(1): p. 63-69.
17. Yeh, C., et al., *Suppression of fatty acid synthase in MCF-7 breast cancer cells by tea and tea polyphenols: a possible mechanism for their hypolipidemic effects.* The pharmacogenomics journal, 2003. **3**(5): p. 267-276.
18. Tsuruta, Y., et al., *Polyphenolic extract of lotus root (edible rhizome of Nelumbo nucifera) alleviates hepatic steatosis in obese diabetic db/db mice.* Lipids in Health and Disease, 2011. **10**(1): p. 1-8.
19. Wu, T., et al., *Oolong tea polysaccharide and polyphenols prevent obesity development in Sprague–Dawley rats.* Food & nutrition research, 2018. **62**.
20. Suk, S., et al., *Gingerenone A, a polyphenol present in ginger, suppresses obesity and adipose tissue inflammation in high-fat diet-fed mice.* Molecular nutrition & food research, 2017. **61**(10): p. 1700139.
21. Cao, W., Y. Dou, and A. Li, *Resveratrol boosts cognitive function by targeting SIRT1.* Neurochemical research, 2018. **43**(9): p. 1705-1713.
22. Csiszar, A., et al., *Resveratrol induces mitochondrial biogenesis in endothelial cells.* American Journal of Physiology-Heart and Circulatory Physiology, 2009. **297**(1): p. H13-H20.

23. Lee, M.-S., et al., *Effects of epigallocatechin-3-gallate on thermogenesis and mitochondrial biogenesis in brown adipose tissues of diet-induced obese mice.* Food & nutrition research, 2017. **61**(1): p. 1325307.
24. Yu, X., et al., *Quercetin attenuates chronic ethanol-induced hepatic mitochondrial damage through enhanced mitophagy.* Nutrients, 2016. **8**(1): p. 27.
25. Wang, X., et al., *TEM observation of ultrasound-induced mitophagy in nasopharyngeal carcinoma cells in the presence of curcumin.* Experimental and therapeutic medicine, 2012. **3**(1): p. 146-148.
26. Baret, P., et al., *Dietary polyphenols preconditioning protects 3T3-L1 preadipocytes from mitochondrial alterations induced by oxidative stress.* The international journal of biochemistry & cell biology, 2013. **45**(1): p. 167-174.
27. Zou, T., et al., *Dietary apple polyphenols promote fat browning in high-fat diet-induced obese mice through activation of adenosine monophosphate-activated protein kinase α.* Journal of the Science of Food and Agriculture, 2020. **100**(6): p. 2389-2398.
28. Han, X., et al., *p-Coumaric acid prevents obesity via activating thermogenesis in brown adipose tissue mediated by mTORC1-RPS6.* The FASEB Journal, 2020. **34**(6): p. 7810-7824.
29. Klaus, S., et al., *Epigallocatechin gallate attenuates diet-induced obesity in mice by decreasing energy absorption and increasing fat oxidation.* International journal of obesity, 2005. **29**(6): p. 615-623.
30. Han, X., et al., *Gentisic acid prevents diet-induced obesity in mice by accelerating the thermogenesis of brown adipose tissue.* Food & Function, 2021. **12**(3): p. 1262-1270.
31. Wang, L., et al., *Green tea polyphenols modulate colonic microbiota diversity and lipid metabolism in high-fat diet treated HFA mice.* Journal of food science, 2018. **83**(3): p. 864-873.
32. Rodríguez-Daza, M.-C., et al., *Berry polyphenols and fibers modulate distinct microbial metabolic functions and gut microbiota enterotype-like clustering in obese mice.* Frontiers in microbiology, 2020. **11**: p. 2032.
33. Liu, J., et al., *Blueberry and cranberry anthocyanin extracts reduce bodyweight and modulate gut microbiota in C57BL/6 J mice fed with a high-fat diet.* European Journal of Nutrition, 2021: p. 1-12.
34. Martín-Peláez, S., et al., *Effect of virgin olive oil and thyme phenolic compounds on blood lipid profile: Implications of human gut microbiota.* European journal of nutrition, 2017. **56**(1): p. 119-131.
35. Anhê, F.F., et al., *A polyphenol-rich cranberry extract protects from diet-induced obesity, insulin resistance and intestinal inflammation in association with increased Akkermansia spp. population in the gut microbiota of mice.* Gut, 2015. **64**(6): p. 872-883.
36. Klinder, A., et al., *Impact of increasing fruit and vegetables and flavonoid intake on the human gut microbiota.* Food & function, 2016. **7**(4): p. 1788-1796.
37. Etxeberria, U., et al., *Reshaping faecal gut microbiota composition by the intake of trans-resveratrol and quercetin in high-fat sucrose diet-fed rats.* The Journal of nutritional biochemistry, 2015. **26**(6): p. 651-660.
38. Zhao, L., et al., *A combination of quercetin and resveratrol reduces obesity in high-fat diet-fed rats by modulation of gut microbiota.* Food & function, 2017. **8**(12): p. 4644-4656.
39. Bartzoka, E.D., et al., *Synthesis of nano-and microstructures from proanthocyanidins, tannic acid and epigallocatechin-3-O-gallate for active delivery.* Green Chemistry, 2017. **19**(21): p. 5074-5091.
40. Hu, B., et al., *Nanochemoprevention by encapsulation of (−)-epigallocatechin-3-gallate with bioactive peptides/chitosan nanoparticles for enhancement of its bioavailability.* Chemical Communications, 2012. **48**(18): p. 2421-2423.
41. Singhal, A., et al., *Colon-targeted quercetin delivery using natural polymer to enhance its bioavailability.* Pharmacognosy research, 2011. **3**(1): p. 35.
42. Bora, A.F.M., et al., *Application of microencapsulation for the safe delivery of green tea polyphenols in food systems: Review and recent advances.* Food Research International, 2018. **105**: p. 241-249.
43. Liang, J., et al., *Applications of chitosan nanoparticles to enhance absorption and bioavailability of tea polyphenols: A review.* Food Hydrocolloids, 2017. **69**: p. 286-292.

44. Flores, G., et al., *In vitro fermentation of anthocyanins encapsulated with cyclodextrins: Release, metabolism and influence on gut microbiota growth.* Journal of Functional Foods, 2015. **16**: p. 50-57.
45. Oehme, A., et al., *Preparation and characterization of shellac-coated anthocyanin pectin beads as dietary colonic delivery system.* Molecular nutrition & food research, 2011. **55**(S1): p. S75-S85.
46. Pham-Hua, D., et al., *Islet encapsulation with polyphenol coatings decreases pro-inflammatory chemokine synthesis and T cell trafficking.* Biomaterials, 2017. **128**: p. 19-32.
47. Zhou, Z., et al., *A novel approach to prepare protein-proanthocyanidins nano-complexes by the reversible assembly of ferritin cage.* Food Science and Technology Research, 2017. **23**(2): p. 329-337.
48. Vitaglione, P., et al., *Human bioavailability of flavanols and phenolic acids from cocoa-nut creams enriched with free or microencapsulated cocoa polyphenols.* British Journal of Nutrition, 2013. **109**(10): p. 1832-1843.
49. Donsì, F., et al., *Encapsulation of bioactive compounds in nanoemulsion-based delivery systems.* Procedia Food Science, 2011. **1**: p. 1666-1671.
50. Isailović, B.D., et al., *Resveratrol loaded liposomes produced by different techniques.* Innovative food science & emerging technologies, 2013. **19**: p. 181-189.
51. Ydjedd, S., et al., *Effect of in vitro gastrointestinal digestion on encapsulated and nonencapsulated phenolic compounds of carob (Ceratonia siliqua L.) pulp extracts and their antioxidant capacity.* Journal of agricultural and food chemistry, 2017. **65**(4): p. 827-835.
52. Yang, Q.-Q., et al., *Nanochemoprevention with therapeutic benefits: an updated review focused on epigallocatechin gallate delivery.* Critical reviews in food science and nutrition, 2020. **60**(8): p. 1243-1264.
53. Tsirigotis-Maniecka, M., R. Gancarz, and K.A. Wilk, *Polysaccharide hydrogel particles for enhanced delivery of hesperidin: Fabrication, characterization and in vitro evaluation.* Colloids and surfaces A: physicochemical and engineering aspects, 2017. **532**: p. 48-56.
